# A novel STAT3 inhibitor W2014-S regresses human non-small cell lung cancer xenografts and sensitizes EGFR-TKI acquired resistance

**DOI:** 10.7150/thno.49600

**Published:** 2021-01-01

**Authors:** Qiyao Zheng, Hui Dong, Jianshan Mo, Yi Zhang, Jie Huang, Shumin Ouyang, Shuo Shi, Kai Zhu, Xinming Qu, Wenhao Hu, Peiqing Liu, Yuanxiang Wang, Xiaolei Zhang

**Affiliations:** 1 Guangdong Key Laboratory of New Drug Design and Evaluation, School of Pharmaceutical Sciences, Sun Yat-Sen University, Guangzhou, 510006, China; 2 Guangdong Key Laboratory of Chiral Molecule and Drug Discovery, School of Pharmaceutical Sciences, Sun Yat-Sen University, Guangzhou, 510006, China; 3 Guangdong Lung Cancer Institute, Guangdong Provincial Key Laboratory of Translational Medicine in Lung Cancer, Guangdong Provincial People's Hospital and Guangdong Academy of Medical Sciences, Guangzhou 510080, China; 4 Innovation Practice Center, Changchun University of Chinese Medicine, Changchun, 130117, China

**Keywords:** STAT3 inhibitor, EGFR-TKI, acquired resistance, W2014-S, non-small cell lung cancer

## Abstract

Constitutive activation of signal transducer and activator of transcription 3 (STAT3) is a common feature in human non-small cell lung cancer (NSCLC). STAT3 plays an important role in cancer progression as a driver oncogene and acquired resistance of targeted therapies as an alternatively activated pathway. W2014-S with pharmacophore structure of imidazopyridine, which was firstly reported to be utilized in STAT3 inhibitor discovery, was screened out as a potent STAT3 inhibitor from a library of small molecules. The aim of this study is to investigate the antitumor activities and mechanisms of W2014-S in NSCLC and effect on epidermal growth factor receptor-tyrosine kinase inhibitors (EGFR-TKIs) resistance* in vitro* and* in vivo*.

**Methods:** SPR analysis, Co-immunoprecipitation, confocal microscope imaging, and luciferase report gene assays were utilized to determine the mechanisms. Cell viability, colonial survival, wound healing, cell invasion assay, human cancer cell xenografts and PDX tumor xenografts were used to determine antitumor activities.

**Results:** W2014-S disrupted STAT3 dimerization and selectively inhibited aberrant STAT3 signaling in NSCLC cell line. W2014-S strongly suppressed proliferation, survival, migration and invasion of lung cancer cells with aberrant STAT3 activation and inhibited the growth of human NSCLC cell xenografts and PDX tumor xenografts in mouse model. Furthermore, W2014-S significantly sensitized resistant NSCLC cell line to gefitinib and erlotinib *in vitro* and enhances the anti-tumor effect of gefitinib in TKI-resistant lung cancer xenografts *in vivo*.

**Conclusions:** Our study has provided a novel STAT3 inhibitor with significant anti-tumor activities in NSCLC and suggests that combination of STAT3 inhibitor such as W2014-S with gefitinib could serve as a promising strategy to overcome EGFR-TKIs acquired resistance in NSCLC patients.

## Introduction

Lung cancer has become the first and third leading cause of cancer deaths in men and women respectively, making it one of the most common and severe cancer worldwide, and non-small cell lung cancer (NSCLC) comprises the vast majority (80-85%) of all lung cancers 1-4. Epidermal growth factor receptor (EGFR), a tyrosine kinase receptor, is the most common driver for NSCLC 5. EGFR tyrosine kinase inhibitors (EGFR-TKIs) have been developed as a first-line therapy for advanced NSCLC patients 1, 6, 7. NSCLC patients treated with EGFR-TKIs show significant improvements in response rates and median progression-free survival compared with standard platinum-based chemotherapy 8, 9. Unfortunately, a high possibility of drug resistance has been reported among NSCLC patients treated with EGFR-TKIs 10-12. Therefore, the management of NSCLC patients who acquire resistance to EGFR-TKI targeted therapy represents a major ongoing challenge.

Somatic activating mutations in EGFR including exon 19 deletion, G719X and L858R, are considered to be correlated with the therapeutic sensitivity of EGFR-TKIs. Except for EGFR mutations accounted for acquired resistance to EGFR-TKIs in NSCLC, aberrantly activated alternative pathways, such as MET amplification and STAT3 hyperactivation, may also contribute to the EGFR-TKIs acquired resistance 13-15. Some studies showed that alternatively activated STAT3 may contribute to drug resistance of EGFR-TKIs 8, 14, 16-18. Numerous clinical studies demonstrated that NSCLC patients who had lower progression-free-survival (PFS) and poor prognosis are characterized by a higher level of STAT3-Y705 phosphorylation 7, 19, 20. Meanwhile, cancer patients with EGFR-TKIs acquired resistance have a high level of phosphorylated STAT3 in some degree 19, 21-24. Although STAT3 can act as downstream of EGFR, the activation of STAT3 in EGFR-TKIs resistance may be EGFR-independent 25-28. These evidences suggested that targeting STAT3 may provide a new strategy to overcome EGFR-TKI acquired resistance in lung cancer.

STAT3 is a key regulator of multiple cellular processes such as proliferation, survival, differentiation, apoptosis, immune function and angiogenesis. Normal STAT3 signaling is tightly controlled in standard cellular response, but constitutive STAT3 activation frequently occurs in a variety of human cancers, especially in lung cancer 25, 29-33. Moreover, STAT3 tyrosine phosphorylation can also be stimulated by EGFR through recruiting to the membrane receptors, except for cytokines, Janus-activated kinases (JAK) and Src family kinases 13, 14, 25, 34. Therefore, targeting STAT3 may not only inhibit NSCLC initiation and progression, but also is able to reverse EGFR-TKIs acquired resistance.

Given its important roles in cancer, STAT3 has been recognized as an attractive cancer therapeutic target. It was reported that STAT3 Y705 phosphorylation and subsequent dimerization of STAT3 are critical steps in the canonical JAK-STAT3 signaling pathway 30, 32, 35. As the Src-homology 2 (SH2) domain plays a pivotal role in STAT3 signaling cascade, targeting STAT3 SH2 domain would prevent the dimerization and transcriptional activity of STAT3 32, 36. Although several approaches have been proposed to develop novel STAT3 inhibitors 37-42, targeting the SH2 domain and disrupting the STAT3 dimerization has been one of the most important strategies. OPB-51602 and OPB-31121 are the promising STAT3 inhibitors in this class. They reached early phase clinical trials and showed promising efficacy in NSCLC patients resistant to TKI therapy 42. Recently, a potent STAT3 degrader SD-36 based on the proteolysis targeting chimera (PROTAC) concept, utilizing a ligand for cereblon/cullin 4A E3 ligase and a peptidomimetic STAT3 SH2 domain inhibitor, was discovered and showed potent anti-tumor activities in blood cancer 39.

Although a lot of efforts have been made to develop specific and potent STAT3 inhibitors, the reported inhibitors still suffer from multiple challenges, such as low specificity, weak binding affinity, low oral bioavailability, poor solubility, structural instability, unfavorable PK profiles, potential severe toxicities, and notably, none of STAT3 inhibitors has been approved for lung cancer therapy in clinic 43.

Constitutive activation of STAT3 has been observed in most cell lines of NSCLC, and plays a pivotal role in tumor progression and acquired resistance to targeted therapies 13, 24, 44, 45. The exploration of STAT3 as a critical pathway in resistance of targeted therapy of oncogene-driven cancers would advance the broader application of STAT3 inhibitors. In this study, W2014, characterized by the core structure of imidazopyridine, was selected out from our in-house compound library. We further synthesized its enantiomers, W2014-S and W2014-R, and found that both of them occupied sub-pockets of the SH2 domain in computational modeling and bound to STAT3 protein with high affinity in surface plasmon resonance (SPR) analysis. While W2014-S exhibited more potent anti-tumor activities in primary studies and selectively inhibited aberrant STAT3 signaling in NSCLC, demonstrating significant anti-tumor activities in cell and animal models. Furthermore, we found that a combination of W2014-S and gefitinib was capable of sensitizing resistant NSCLC cells to gefitinib *in vitro* and *in vivo*. Hence, our studies discovered a novel STAT3 inhibitor W2014-S with potent antitumor activities in NSCLC and suggested that the combination of STAT3 inhibitor with gefitinib could serve as a promising strategy to overcome EGFR-TKIs resistance in NSCLC patients.

## Materials and Methods

### Chemical methods and synthetic route

Synthetic methods and Routes are described in detail in Supplementary Information*.*

### Cells culture

The human non-small-cell lung cancer (NSCLC) cell line A549 and the human embryonic kidney (HEK) cell line 293T were purchased from American Type Culture Collection (ATCC, VA, USA). The NSCLC cell lines PC-9 and PC-9/GR were purchased from European Collection of Authenticated Cell Cultures (ECACC, London, UK). A549, PC-9 cells were cultured in RPMI-1640 (Gibco, Carlsbad, CA, USA) containing 10% fetal bovine serum (FBS, Gibco, Carlsbad, CA, USA) and 1% penicillin-streptomycin (P.S., Gibco, Carlsbad, CA, USA), 293T cells were cultured in DMEM (Gibco, Carlsbad, CA, USA) containing 10% FBS and 1% PS. Gefitinib-resistant PC-9/GR cells were obtained by continuously culturing the cells in gefitinib (800 ng/mL) and cultured in DMEM with 10% FBS. All cell lines were cultivated at 37 ℃ under 5% CO_2_.

### Reagents and antibodies

Gefitinib was provided by Selleck (ZD1839, Houston, TX, USA). Erlotinib (#S7786) was also provided by Selleck (Houston, TX, USA). The primary antibodies STAT3 (#12640), pY705-STAT3 (9145S), pY701-STAT1 (#9167), STAT5 (#25656) , phospho694-STAT5 (#9351), EGFR (4267S), phospho-EGFR (3777S), JAK2 (#3230), phospho-JAK2 (#3771), AKT (#4685), p-AKT (#4060), c-Myc (#5605), and c-Caspase-7 (#9491) were from Cell Signaling Technology (Danvers, MA, USA). STAT1 (10144-2-AP) was from Proteintech (Wuhan, Hubei, China). Bcl-2 (sc-7382), Bcl-xL(sc-8392) and β-actin (sc-47778) were from Santa Cruz (Dallas, TX, USA). The secondary antibodies were HRP-conjugated anti-rabbit IgG, anti-mouse IgG (Abcam, Cambridge, UK). EGF (AF-100-15-500) was provided by PEPROTECH (Suzhou, China).

### Plasmids and molecular cloning

The STAT3-dependent luciferase reporter, pGL3-STAT3, contained seven copies of the STAT3-specific binding sequence (AATCCCAGAA) in the C-reactive protein gene promoter, and drove firefly luciferase expression. The new construct was confirmed by sequencing. For luciferase studies, 293T cells or A549 cells in 96-well plates were transiently co-transfected with the appropriate plasmids (10 ng TKRL as internal control vector for normalizing and 50 ng of pGL3-STAT3) using Lipofectamine 2000 (11668500, Invitrogen, Carlsbad, CA, USA) which was following the manufacturer's protocol. After 24 h, HEK-293T cells were treated with 0-1 μM W2014-S for 24 h, and stimulated with IL-6 (100 ng/mL, PeproTech) for 1 h or EGF (50 ng/mL, PeproTech) for 30 min. Subsequently, cytosolic extracts were prepared for dual-luciferase reporter assay.

### RNA interference

PC-9/GR cells were seeded into 6-well plates. After incubating for 18-24 h, cells grew to 60-70%. According to the manufacturer's protocol of DharmaFECT (T-2001-03, Dharmacon, USA), cells were transfected with negative control or siRNA targeting STAT3. For STAT3 interference, the sequence of two sense strands of siRNA targeting STAT3:

STAT3-1: 5'-UCCAGUUUCUUAAUUUGUUGACGGGUC-3'; STAT3-2: 5'-AUAGUCCUAUCUUCUAUUUGGAUGUCA-3';

The sequence of two antisense strands of siRNA targeting STAT3 as follows:

STAT3-1: 5'-GACCCGUCAACAAAUUAAGAAACUGGA-3'; STAT3-2: 5'-UGACAUCCAAAUAGAAGAUAGGACUAU-3'.

A nonspecific oligonucleotide without complementary to any human gene was used as negative control. All above siRNAs were synthesized by Sangon Biotech (Guangdong, China).

### CRISPR/Cas9-mediated knockout of STAT3

To knockout the STAT3 gene in A549 cells using CRISPR/Cas9 system, the lentiCRISPRv2 vector (gift from Professor Junjian Wang, Sun Yat-Sen University, China) was used. An optimized guide RNA (gRNA) targeting the exon2 of STAT3 was designed in an online CRISPR design tool (http://crispor.tefor.net/). Then the guide RNA (synthesized by Sangon Biotech) inserted into lentiCRISPRv2 vector and the recombinant plasmid (sgSTAT3) was transfected and amplified in *E.coli* JM109. The confirmed and purified sgSTAT3 plasmid was transfected into A549 cells using lentivirus, after 48h post-transfection, cells were selected by introducing into 3 μg/mL puromycin for 2 weeks. Then the puromycin-resistant cells were planted into 96-wells plate with a density of one single cell per well and expanse to select the STAT3-konckout cells. The STAT3-konckout cells isolated from the single-colony were verified by western blotting analysis. The gRNA used to knockout STAT3 as follows,

Forward primer: 5'- CACCGCAGCTTGACACACGGTACC-3'; Reverse primer: 5'- AAACGGTACCGTGTGTCAAGCTGC-3'.

### Cell viability assay

Cell proliferation was measured using the Cell Counting Kit-8 (CCK-8, B34304, Bimake, USA). A549 (2 × 10^3^/100 μL/well), PC-9 (3 × 10^3^/100 μL/well) or PC-9/GR cells (3 × 10^3^/100 μL/well) were plated into each well of 96-well plates (Costar, USA). After incubating 24 h, several concentrations of W2014-S, W2014-R, gefitinib, and/or culture supernatants were added to each well, and incubation was continued for another 72 h. Then, 10 μL of CCK-8 was added to each well, and cells were incubated for 1-4 h until the color of untreated controls turned to orange. The absorbance (A) was measured by a microplate reader (FLUOstar Omega-ACU, USA) at test and reference wavelengths of 450 nm. The percentage of growth was calculated as Cell viability (%) = [A (Compound +) - A (Blank) ] / [A (Compound -) - A (Blank) ] × 100%. Cell viability meant proliferation of cells. Each experiment was done at least in triplicate, and thrice independently.

### Wound healing assay for migration

The motility of cells was measured by wound healing assay. Cells were seeded into 6-well plates and cultured to 90-100% confluent after 24 h. Wounds were made in each well of 6-well plates using pipette tips. Subsequently, cells were treated with W2014 or gefitinib and allowed to migrate into the denuded area over 48 h. The migration of cells was visualized under a microscope (4 ×, Nikon, Japan) and photographed in different time points (0 and 48 h).

### Transwell cell invasion assay

Transwell invasion assays were performed as previously reported 37 using Transwell chamber (Costar, USA). Cells (3 × 10^5^/300 μL) were seeded into the top transwell chamber with RPIM-1640 culture medium containing 10% FBS, and the bottom chamber was filled with 500 μL medium. After 12 h, the culture medium in the top chamber was changed to RPIM-1640 without FBS and added W2014 or gefitinib. After another 12 h, the invading cells were fixed and stained by crystal violet solution. The cells were photographed under microscope (10 ×, Nikon, Japan) and counted from five randomly selected fields. Data from triplicates experiments were pooled and plotted as shown.

### Western blot and immunoprecipitation

Cells or tumor tissue were lysed with RIPA buffer (Beyotime, Shanghai, China) supplemented with protease inhibitors (Beyotime, Shanghai, China) and phosphatase inhibitors (Bimake, USA). Lysates were denatured by heating for 5 min at 99 °C and loaded on 4-10% SDS-polyacrylamide gel electrophoresis. Then the proteins were transferred to polyvinylidene fluoride (PVDF) membranes (Millipore, USA). The membranes were blocked and probed with primary antibodies and secondary HPR-conjugated antibodies. At last the membranes were detected by chemiluminescence (Tanon, Shanghai, China). For co-immunoprecipitation experiments, 293T cells were plated and grew to about 80% confluency, then co-transfected with plasmids of HA-STAT3 and Flag-STAT3 using Lipofectamine 2000. After 24 h, cells were treated with different concentrations of W2014-S for following 24 h, and stimulated with IL-6 (100 ng/mL) for 1 h. Cells were lysed with IP RIPA buffer (Beyotime, Shanghai, China) supplemented with protease inhibitors (Beyotime, Shanghai, China) and phosphatase inhibitors (Bimake, America), and lysates were cultured with Anti-flag Affinity Gel (B23102, Bimake, USA) overnight at 4 ℃. The gel was washed by PBST for three times and added 1×loading buffer and denatured by heating for 5 min at 99 ℃. Then the proteins were resolved on SDS-PAGE, transferred to PVDF membranes and analysed with immunoblotting.

### Immunofluorescence

A549 cells were cultured into confocal dishes (Costar, USA). After 24 h, W2014-S was added into dishes. After another 3 h, cells were fixed with 4% paraformaldehyde in PBS for 10 min at room temperature. Then cells were permeabilized with 0.3% Trition X-100 for 5 min at room temperature, following by incubating with primary antibody overnight at 4 ℃. Next, cells were incubated with Alexa-Fluor-conjugated secondary antibodies for 1 h in the dark. The cells were subsequently stained with DAPI (40728ES50, Yeasen, Shanghai, China). Images were captured by Laser Scanning Confocal Microscope FV3000 (Olympus, Japan). *Z*-stacks were collected with a spacing of 1 μm. Antibody and dilutions used in the studies were: pY705-STAT3 (9145S, Cell Signaling Technology, 1:100), Alexa Fluor 488 Conjugate (1:5000, #4412, Cell signaling Technology, USA). The fluorescence intensity profile along the *Z*-axis from confocal *Z*-stacks was shown. The fluorescence intensity of nuclear-localized p-STAT3 was quantified using the confocal software to define the area of the selected region of interest based on the nuclear DAPI signal. Each experiment was done in at least 3 independent experiments.

### Cell apoptosis assay

Cell apoptosis induced by gefitinib or W2014-S was detected with an Annexin V-FITC Apoptosis Detection Kit I (BestBio, Shanghai, China) in accordance with the manufacturer's protocols. Briefly, aliquots of 2 × 10^5^ cells were seeded into 6-well plates and incubated for 24 h at 37 °C. On the following day, gefitinib and/or W2014-S, diluted in culture media, were added to the dishes and incubated for an additional 72 h. After trypsinization and gently washing cells once with medium, the cells were washed twice with cold PBS. The precipitation was resuspended by 400 μL of 1 × binding buffer, and then resuspended cells were transferred into new 1.5 mL EP tubes. Then 3 μL of Annexin V-FITC and 5 μL of propidium iodide were added to the resuspended cells with further incubation at room temperature for 15 min in the dark. The analysis was done on Guava easyCyte (USA) and FlowJo 7.6 software.

### Surface plasmon resonance analysis

Biacore 8K and Biacore Insight Evaluation software were used to analyze the interaction between the agents and the protein full-length wild-type STAT3 to detect the binding affinity. Purified STAT3 (100 μg/mL) without Tris and glycerin was injected onto the CM5 Chip (GE, USA) for immobilization. Several concentrations of W2014-S or W2014-R dissolved in running buffer (1×PBS with filtration, 0.01% DMSO) were flown over the chip to produce response signals. The kinetics and affinities were calculated by the Biacore Insight Evolution software, and the results were determined as the binding affinity (*K_D_*).

### Docking analysis

Briefly, Maestro 11.1 software (available from Schrödinger, Inc.) was employed to dock small molecule 3D structures from NCI Plated Set to the ApY*LK site derived from the X-ray crystal structure of the STAT3 (PDB code: 1BG1) dimer. Schrödinger's Protein Preparation Wizard was used in the preparation of the protein structure and Schrödinger's LigPrep was used to prepare molecules for docking. Schrödinger's Receptor Grid Generation was used for the generation of grid files. Grid box was prepared at its SH2 domain, and Schrödinger's Ligand Docking was used for docking of the protein structure and ligand. Protein was considered rigid and small molecules were flexible during docking process. The XP extra precision was chosen as the vital docking parameters. Schrödinger's Protein Surface Analyzer was used to analyze the protein structure and color-coding based on electronegativity.

### Tumor xenografts

The animal procedures were approved by the Research Ethics Committee of Sun Yat-sen University (SYSU-IACUC-2019-000110) and conducted following the Guide for the Care and Use of Laboratory Animals. Four-week-old nude mice (male, weighing 16-17g, SPF grade, certification No. SYXK (Guangdong) 2016-0112) achieved from the Experimental Animal Center of Sun Yat-sen University (Guangdong, China). To establish cell xenograft model, nude mice were injected subcutaneously with A549 cells (2×10^6^) or PC-9/GR cells (4×10^6^). For PDX model, the PDX sample was from The Jackson Laboratory. The model ID is TM00192/LG0567F. For this patient, the primary site is lung, the initial and diagnosis and final diagnosis is lung adenocarcinoma. The stage/grade is AJCC IB/grade 3. The sex is male, age is 57, race is Asian or Pacific islander. The sample type is surgical resection. Sample from passage P0 has a TMB score of 7.27 and there is very strong morphologic fidelity between patient tumor and P0. The tumor sample were cut into approximately 1 mm^3^, and the tissue was pushed under skin of mice by TROCHAR. When tumor volumes reached approximately 100 mm^3^, mice were randomly distributed into groups of 7 mice. Mice were received either vehicle control, gefitinib alone, W2014-S alone or gefitinib and W2014-S together. Gefitinib was dissolved in PBS containing 15% Polyoxyl 35 Hydrogenated Castor Oil and administered every day by oral gavage (50 mg/kg). W2014-S was suspended in PBS containing 15% Polyoxyl 35 Hydrogenated Castor Oil and administered once a day by intraperitoneal injection (15 mg/kg). Tumor size was measured every two days by vernier caliper. The average tumor volume in each group was calculated based on the equation for a prolate spheroid (tumor volume = (short-diameter)^2^ × large-diameter × π / 6 ) and expressed in mm^3^.

### Acute toxicity and pharmacokinetic study

KM mice (4-5 weeks of age, weighing 18-22g, both male and female) for acute toxicity experiment were provided from the Experimental Animal Center of Sun Yat-sen University (Guangdong, China) and were housed in a specific pathogen-free facility. To test the acute toxicity, 40 mice were randomly distributed into 4 groups, and treated with W2014-S at 100 mg/kg, 300 mg/kg, and 500 mg/kg by intraperitoneal injection. Each group have 10 mice (half male and half female). All of the mice were observed for 14 days after treatment, body weight of mice was measured every two days, recorded the activities of mice and counted the quantities of mice died. All animal experiments were approved by the Research Ethics Committee of Sun Yat-sen University (SYSU-IACUC-2020-000312).

W2014-S was given to SD rats (male, 230-260g, n = 6) by gavage and intravenous administration. The rats were fasted for 12 h before administration and remained fasting for 2 h. For oral gavage, W2014-S was dissolved in DMSO/0.5% HPMC (5/95, v/v/), and W2014-S was dissolved in DMSO/EtOH/PEG300/0.9% NaCl (5/5/40/50, v/v/v/v) for intravenous administration. The pharmacokinetic (PK) profiles were next evaluated in SD rats.

### Statistical analysis

Statistical analysis was performed on mean values using Prism (GraphPad Software, USA). The significance of differences between groups was determined via the unpaired t-test as ^*^P<0.05, ^**^P<0.01, ^***^P<0.001.

## Results

### Discovery of a potent and selective STAT3 inhibitor W2014

**Figure 1:**
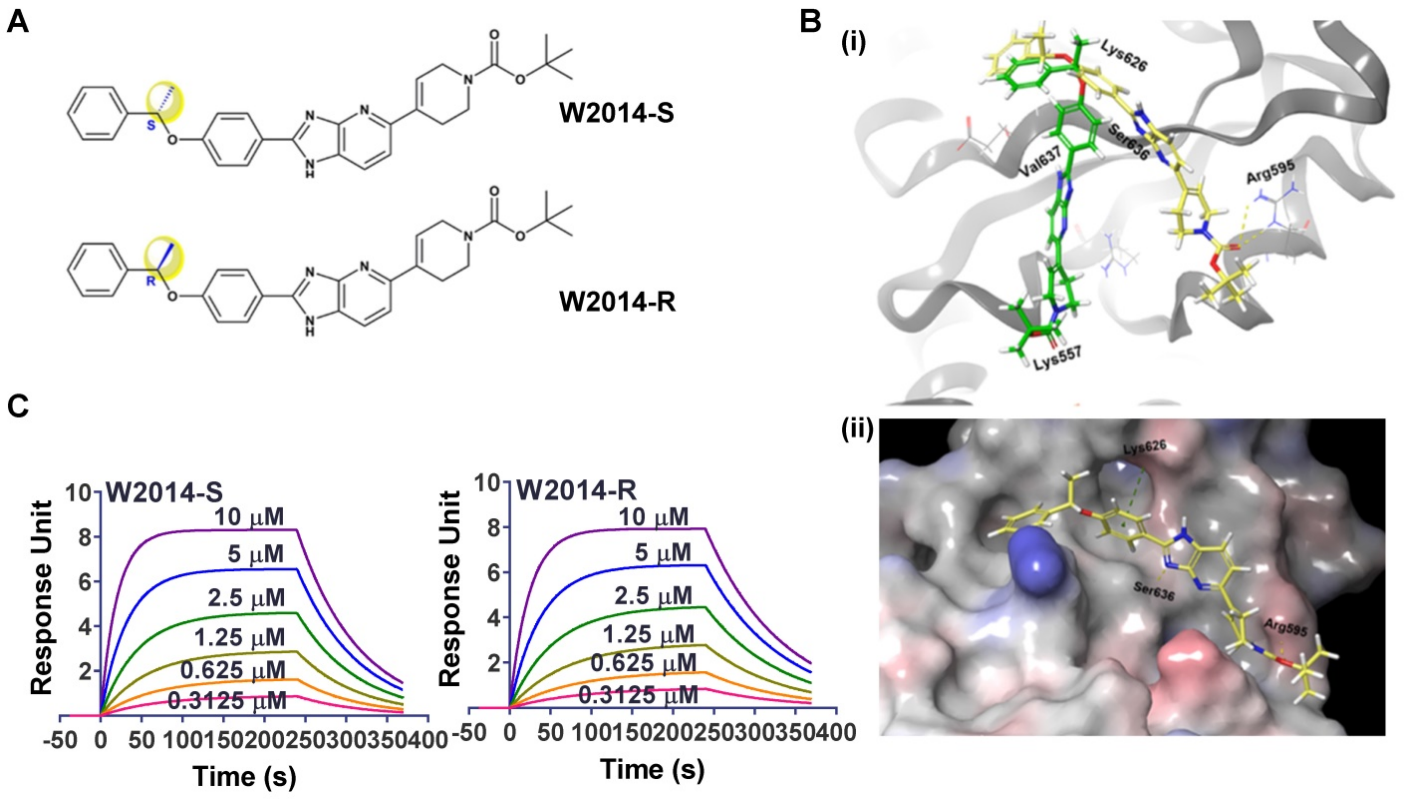
The interactions between W2014-S/R with STAT3 protein. **(A)** Chemical structure of W2014-S and W2014-R. **(B)** Computational modeling of W2014-S and W2014-R binding to STAT3. (i) The yellow molecule represents W2014-S and the green one represents W2014-R. (ii) W2014-S occupies sub-pockets in SH2 domain of STAT3. **(C)** SPR analysis of the binding of W2014-S/R to STAT3.

W2014 was screened out as a potent STAT3 inhibitor from an in-house library of small molecules Figure S1. The core structure of W2014 is imidazopyridine with one asymmetric center, of which pharmacophore was first utilized to design STAT3 inhibitors. W2014-S was S configuration of this chiral molecule, and W2014-R was the R configuration (Figure 1A). The chemical synthetic route is as shown in Figure S13-21. There are solvent-accessible subpockets within the STAT3 SH2 domain-binding surface that can be accessed by STAT3 inhibitors. To determine the potential binding mode of W2014 to STAT3 protein, we performed docking studies as described in Methods Section. W2014-S and W2014-R bind to different sites on STAT3 SH2 domain in modeling, in spite of the same chemical formula (Figure 1B). W2014-S occupied the phosphotyrosine-binding site of STAT3 and formed hydrogen bonds with Arg595 and Ser636, and cation-π interaction with Lys626. XP GScore suggested W2014-S may bind to STAT3 SH2 domain with higher affinity than W2014-R. While surface plasmon resonance (SPR) analysis suggested that two compounds may have similar affinities with STAT3 protein. W2014-S and W2014-R bind to wild-type STAT3, with *K_D_* of 3.64 μM and 3.39 μM, respectively (Figure 1C). It suggested that these two compounds have similar affinities with STAT3 protein. However, W2014-S and W2014-R showed distinct antitumor activity in cell model in following study, and we hypothesized that the distinct binding sites and binding model may affect the antitumor effect.

### W2014-S strongly suppressed proliferation, survival, migration and invasion of lung cancer cells with aberrantly-active STAT3

**Figure 2:**
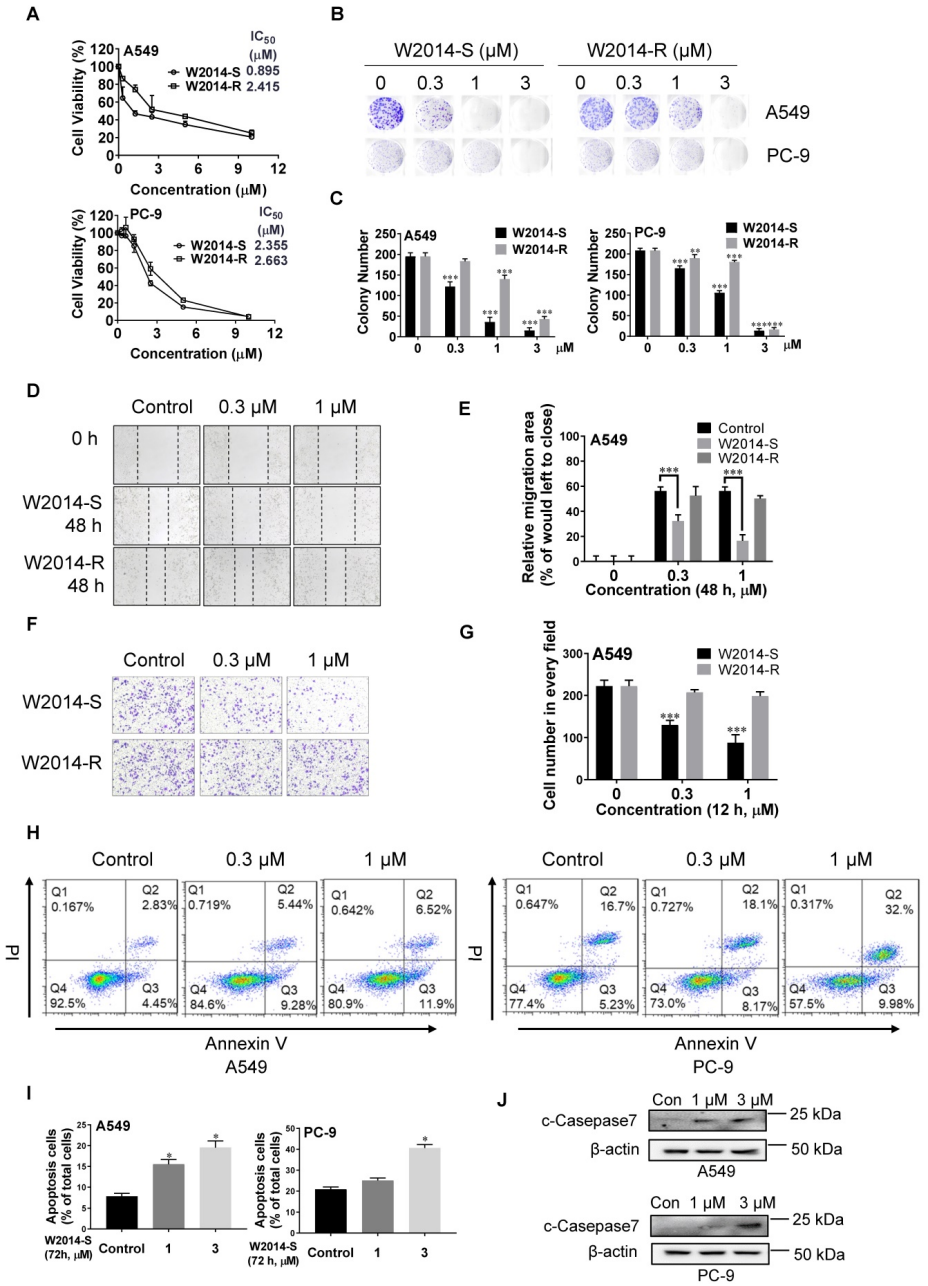
** Antitumor effects of W2014-S and W2014-R against malignant cells harboring aberrantly-active STAT3 *in vitro****.* NSCLC cell lines A549 and PC-9 with constitutive STAT3 were treated with W2014-S or W2014-R at different concentrations (0, 0.3, 1, 3 μM). **(A)** Cell proliferation and IC50 values were measured in A549 and PC-9 cells at 72 h. Each point represented the mean ± SEM, n=3.** (B-C)** Numbers of colonies of A549 and PC-9 cells were detected by colony formation assay. Data were expressed as mean ± SEM, n=3. ^*^P < 0.05, ^**^P < 0.01, *^**^P < 0.001. **(D-E)** Cell migration of A549 cells was detected by wound healing assay. Data were expressed as mean ± SEM, n=3. ^*^P < 0.05, ^**^P < 0.01, *^**^P < 0.001. **(F-G)** Cell invasion of A549 cells was measured by transwell cell invasion assay. **(H-I)** A549 and PC-9 cells were treated with W2014-S for 72 h and apoptosis was measured by flow cytometry. The figures were modified due to the lower font size of labels in original picture. PI means propidium iodide. Data were expressed as mean ± SEM, n=3. ^*^P < 0.05, ^**^P < 0.01, *^**^P < 0.001. **(J)** Western bolt was used to detect the expression of c-Caspase-7 protein.

Lung cancer with constitutively activated STAT3 is one of the most malignant cancers. NSCLS cell lines A549 and PC-9 with high level of phosphorylated STAT3 were treated with W2014-S and W2014-R to determine the anti-tumor effect in cell model. W2014-S and W2014-R inhibited the cell proliferation dose-dependently with IC_50_ values of 0.89 μM and 2.42 μM in A549 cell line, and of 2.36 μM and 2.66 μM in PC-9 cell line, respectively (Figure 2A). W2014-S and W2014-R significantly suppressed the colony formation at as low as 0.3 μM concentration in A549 and PC-9 cell lines in colony survival assay (Figure 2B). W2014-S showed stronger inhibition than W2014-R on cell proliferation and colony survival, especially in A549 cell line.

**Figure 3:**
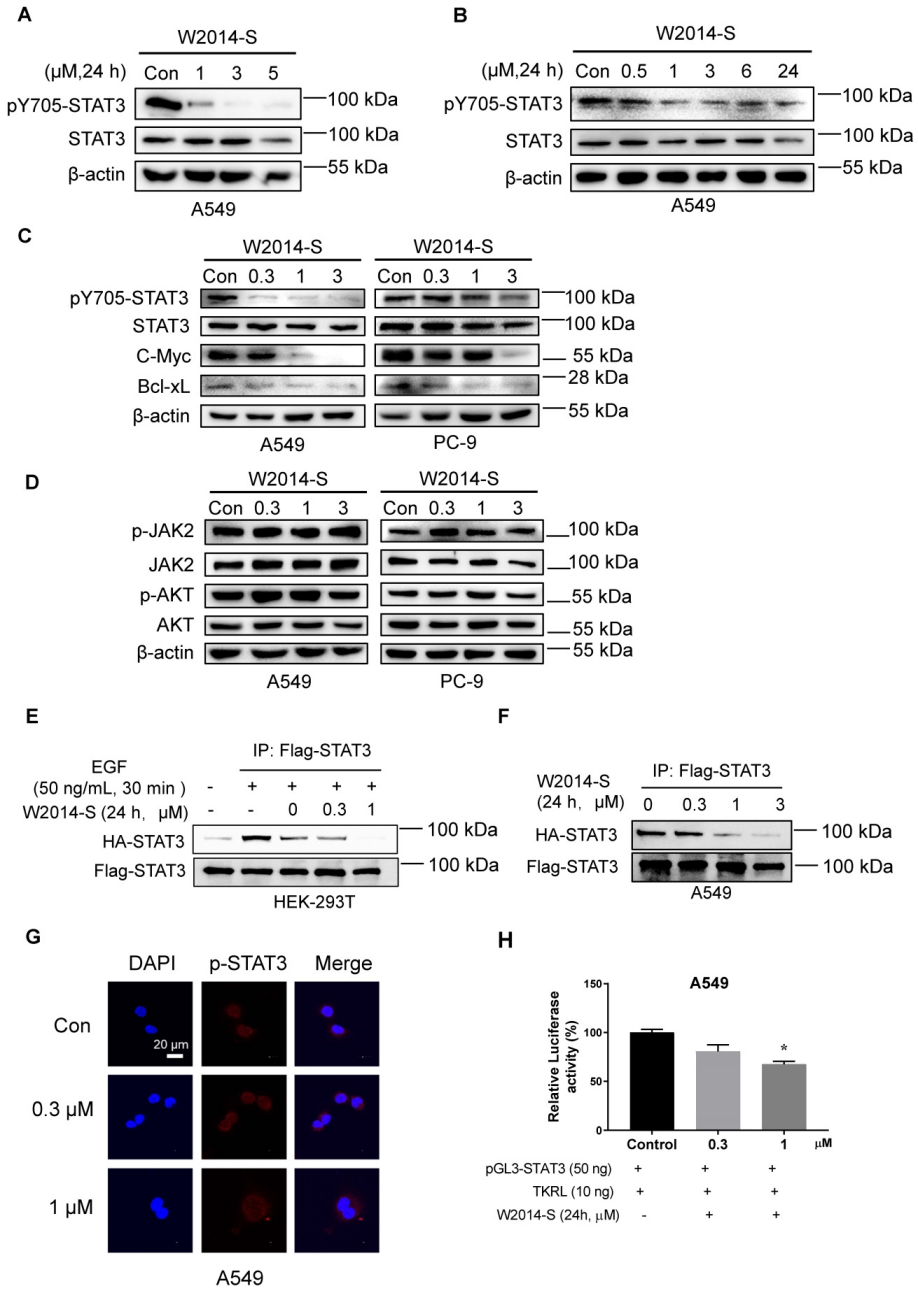
** W2014-S disrupted STAT3 dimerization and significantly inhibited STAT3 signaling. (A)** Western blot was used to detect the expression of STAT3 and pY705-STAT3 in A549 cells treated with W2014-S at 0, 1, 3 and 5 μM for 24 h.** (B)** The expression of STAT3 and pY705-STAT3 in A549 cells treated with 1 μM W2014-S for 0, 0.5, 1, 3, 6 and 24 h. **(C)** The protein changes of pY705-STAT3, STAT3, c-Myc, Bcl-2 and Bcl-xL in A549 and PC-9 cells treated with W2014-S at 0, 0.3, 1, 3 μM for 24 h.** (D)** The expression of pJAK2, JAK2, pAKT and AKT in A549 and PC-9 cells treated with W2014-S at 0, 0.3, 1, 3 μM for 24 h. **(E)** Immunoprecipitation was used to detect the dimerization of STAT3 in 293T cells treated with W2014-S at 0, 0.3, 1 μM for 24 h and stimulated with 50 ng/mL EGF for 30 min.** (F)** Immunoprecipitation was used to detect the dimerization of STAT3 in A549 cells treated with W2014-S at 0, 0.3, 1 μM for 24 h. **(G)** Immunofluorescence images represented the translocation of pY705-STAT3 and scale bar was 20 μm. **(H)** Dual-luciferase reporter assay was used to measure the translational activity of A549 cells treated with W2014-S at 0, 0.3, 1 μM for 24 h. Data were expressed as mean ± SEM, n=3. ^*^P < 0.05, ^**^P < 0.01, *^**^P < 0.001.

As cancer cell migration and invasion contribute to tumor metastasis and patient mortality, W2014-S and W2014-R were further investigated for their effect on cancer cell migration and invasion. Wound healing assay showed that W2014-S remarkably inhibited the migration of A549 in a dose-dependent manner, but W2014-R showed slightly inhibition (Figure 2D-E). Transwell cell invasion assay results suggested that the invasion of A549 cells was suppressed by 1 μM W2014-S, but not W2014-R under the same condition (Figure 2F-G). To examine whether the proliferative inhibition by W2014-S was attributed to apoptosis, A549 and PC-9 cell lines were treated by W2014-S for 72 h to determine the apoptotic cells by flow cytometry. As shown in Figure 3H and 3I, W2014-S induced apoptosis in A549 and PC-9 cells even at 1.0 μM. Cleavage of caspase-7, one of the hallmarks of apoptosis, was induced by W2014-S in A549 and PC-9 (Figure 2J). Taken together, W2014-S exhibited more potent anti-tumor activities than W2014-R and was selected for further studies. We also knocked down or knock out STAT3 in A549 cells to examine the anti-cancer actions of W214-S in non-small cell lung cancer. The anti-cancer activities of W2014-S was significantly attenuated in A549 cells in which STAT3 was knocked down by siRNA interference Figure S6A-C). And then we knockout the STAT3 gene in A549 cells using CRISPR/Cas9 system with lentiCRISPRv2 vector. We also observed that anti-cancer activities of W2014-S was significantly attenuated in A549 cells in which STAT3 was knocked out by Crispr/cas9 system (Figure S6D-F).

### W2014-S disrupted STAT3 dimerization and inhibited STAT3 signaling in NSCLC cells

Constitutively activated STAT3 promoted the expression of its target genes to regulate multiple cellular processes including proliferation, survival, migration, invasion, and anti-apoptosis. To determine whether antitumor activity of W2014 depending on STAT3 signaling inhibition, we carried out the following studies. A549 cells were treated with W2014-S and W2014-R for 24 h to examine the effect on STAT3 signaling. W2014-S was found to inhibit the phosphorylation level of STAT3 in a dose dependent manner, and more potent than W2014-R (Figure 3A and Figure S8A-B). Time course study showed that the phosphorylation of STAT3 at Y705 was inhibited after 1 h treatment of W2014-S (Figure 3B). Subsequently, expression of STAT3 downstream genes including Bcl-2, Bcl-xL and c-Myc was inhibited by W2014-S dose-dependently in A549 and PC-9 cells (Figure 3C). To evaluate the specificity of W2014-S, we examined the effect of W2014S on STAT1 and STAT3 activation in A549 and PC-9 cell line. We found that W2014-S has no obvious effect of STAT1 and STAT5 activation (Figure S3A-B). While W2014-S had little or no effect on the protein level of phosphorylated JAK2 and total JAK2 (Figure 3D), which is a upstream protein of STAT3. Moreover, phosphorylation of AKT and total AKT, which is regulated by PI3K but not STAT3, was not affected by W2014-S (Figure 3D). These results indicated that W2014-S inhibited STAT3 activation without affecting upstream or other kinases. The western blotting results were quantified as shown in Figure S8C-M.

The dimerization of STAT3 monomers is critical for the translocation of STAT3 into nucleus to act function as a transcriptional factor. STAT3 dimerization is formed through two reciprocal phosphotyrosine-SH2 domain binding interactions. To examine the effect of W2014-S on the STAT3 dimerization, we generated a cell model which could measure direct STAT3-STAT3 dimerization in intact cells by co-transfecting HA-tagged STAT3 and Flag-tagged STAT3 into HEK-293T cell line. Subsequently, co-immunoprecipitation studies were performed to verify whether the intracellular binding of two full-length STAT3 proteins was inhibited by W2014-S. HEK-293T cells co-transfected with Flag-STAT3 and HA-STAT3 were treated by W2014-S for 24 h and 50 ng/mL EGF for 30 min before harvesting. As shown in Fig. 3E, W2014-S inhibited the binding of HA-STAT3 and Flag-STAT3 in intact cells even at 0.3 μM. Moreover, we co-transfected HA-STAT3 and Flag-STAT3 into A549 cells with constitutively active STAT3 and performed Co-IP studies. We found that dimerization of HA-STAT3 and Flag-STAT3 was inhibited by W2014-S in a dosage-dependent manner (Figure 3F).

STAT3 phosphorylation and dimerization is required for translocation of STAT3 into nucleus. Furthermore, nuclear translocation studies stated that the accumulation of pY705-STAT3 in the nucleus was decreased by W2014-S with 3 h treatment (Figure 3G). Luciferase reporter studies showed that STAT3-dependent luciferase reporter activity induced by IL-6, was dose-dependently inhibited by W2014-S (Fig. S4. We also performed luciferase reporter gene assay in A549 cells and STAT3 transcriptional activity was inhibited by W2014-S (Figure 3H).

### W2014-S inhibited the growth of human lung cancer cell xenografts and PDX model

**Figure 4:**
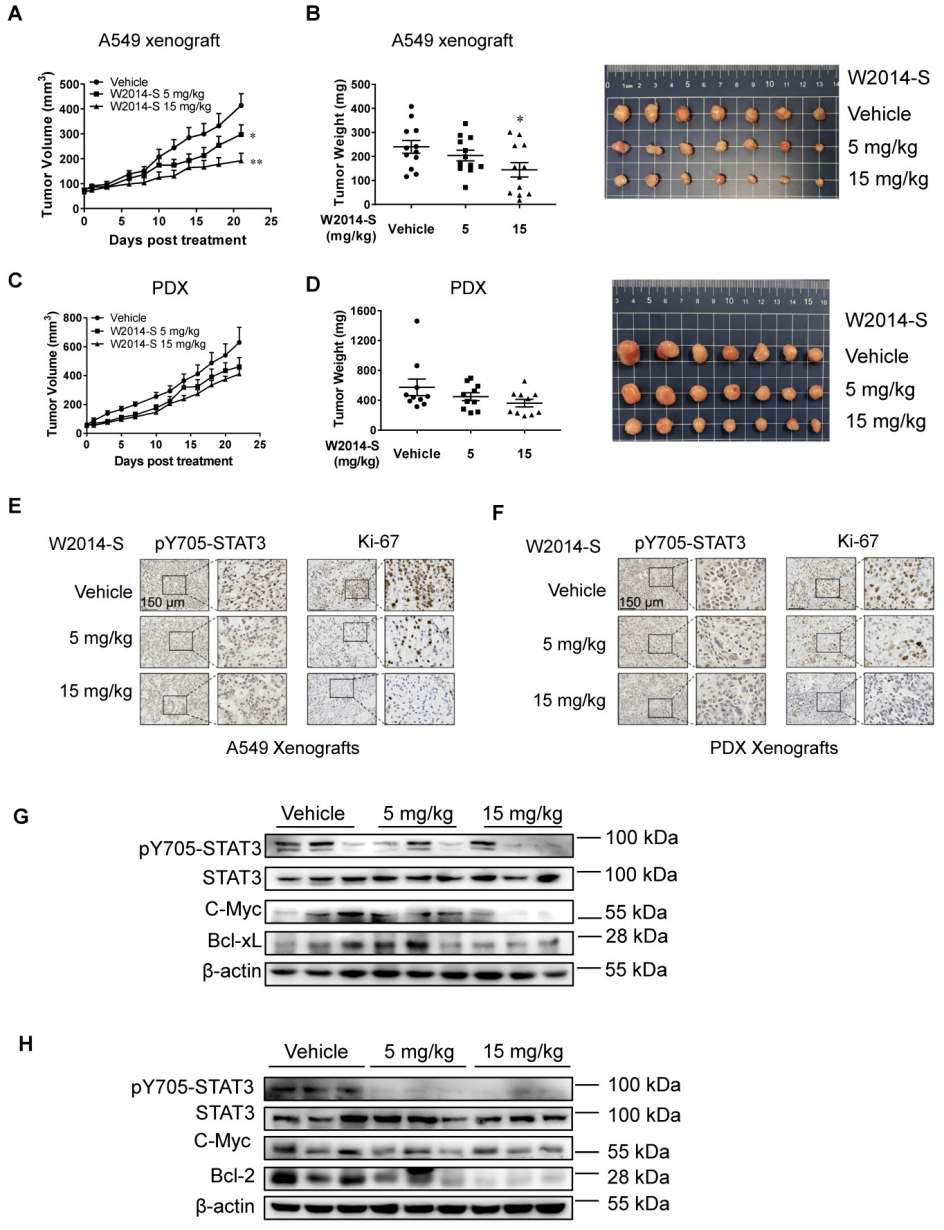
** The anti-tumor effects of W2014-S in human lung cancer cell xenografts and PDX models* in vivo.***Nu/nu mice were bearing A549 xenografts or PDX and treated with W2014-S (i.p. 5 or 15 mg/kg/day) for 21 d. **(A)** Tumor volume of A549 xenografts. Data were expressed as mean ± SEM, n=6. ^*^P < 0.05, ^**^P < 0.01, *^**^P < 0.001. **(B)** Tumor weights and size of A549 xenografts. Data were expressed as mean ± SEM, n=6. ^*^P < 0.05, ^**^P < 0.01, *^**^P < 0.001. **(C)** Tumor volume of PDX. Data were expressed as mean ± SEM, n=6. ^*^P < 0.05, ^**^P < 0.01, *^**^P < 0.001. **(D)** Tumor weights and size of PDX. Data were expressed as mean ± SEM, n=6. ^*^P < 0.05, ^**^P < 0.01, *^**^P < 0.001.** (E-F)** The protein levels of pY705-STAT3 and Ki-67 of A549 xenografts and PDX of mice was detected by IHC and scale bar was 150 μm. **(G-H)** The changes of pY705-STAT3, STAT3, c-Myc, Bcl-xL and Bcl-2 levels of A549 xenografts and PDX were measured by western blot.

To further demonstrate the therapeutic efficacy of W2014-S, subcutaneous mouse xenografts of NSCLC cell line (A549) and lung cancer patient-derived xenograft (PDX) model harboring aberrantly active STAT3 were utilized to evaluate the anti-tumor effect. W2014-S significantly inhibited growth of human lung cancer (A549) xenografts (Figure 4A) and PDX xenografts (Figure 4C) following 21-day of treatment with a daily 5 mg/kg or 15 mg/kg schedule by intraperitoneal injection. The tumor volumes and tumor weights were dose-dependently decreased in 5 mg/kg and 15 mg/kg groups for A549 xenograft tumors (Figure 4B) and PDX xenograft tumors (Figure 4D). Meanwhile, there were no significant changes in body weights, or obvious signs of toxicity, such as loss of appetite, decreased activity, or lethargy during treatment Figure S9 and S10).

The tumor mitotic index (Ki-67) and STAT3 signaling in tumor tissues were evaluated by immunohistochemistry (IHC) and western blotting. As shown in Figure 4E and 4F, expression of Ki-67 was significantly reduced by W2014-S compared with the vehicle group in both mouse models, indicating that tumor proliferation and progression was suppressed after W2014-S administration. IHC analysis of tumor xenografts showed the level of pY705-STAT3 was decreased in W2014-S treated group (Figure 4E-F). Expression of pY705-STAT3, as well as STAT3 downstream genes Bcl-2, c-Myc and Bcl-xL were also examined by western blotting. W2014-S significantly suppressed the level of pY705-STAT3 and subsequently downregulated expression of STAT3 targeting genes in human lung cancer cell xenografts and PDX tumor xenografts as shown in Figure 4G-H. Taken together, these data suggested that W2014-S significantly suppressed excessive STAT3 signaling as well as tumor growth in mouse models.

### W2014-S significantly enhanced gefitinib sensitivity in sensitized gefitinib-resistant cell line

**Figure 5:**
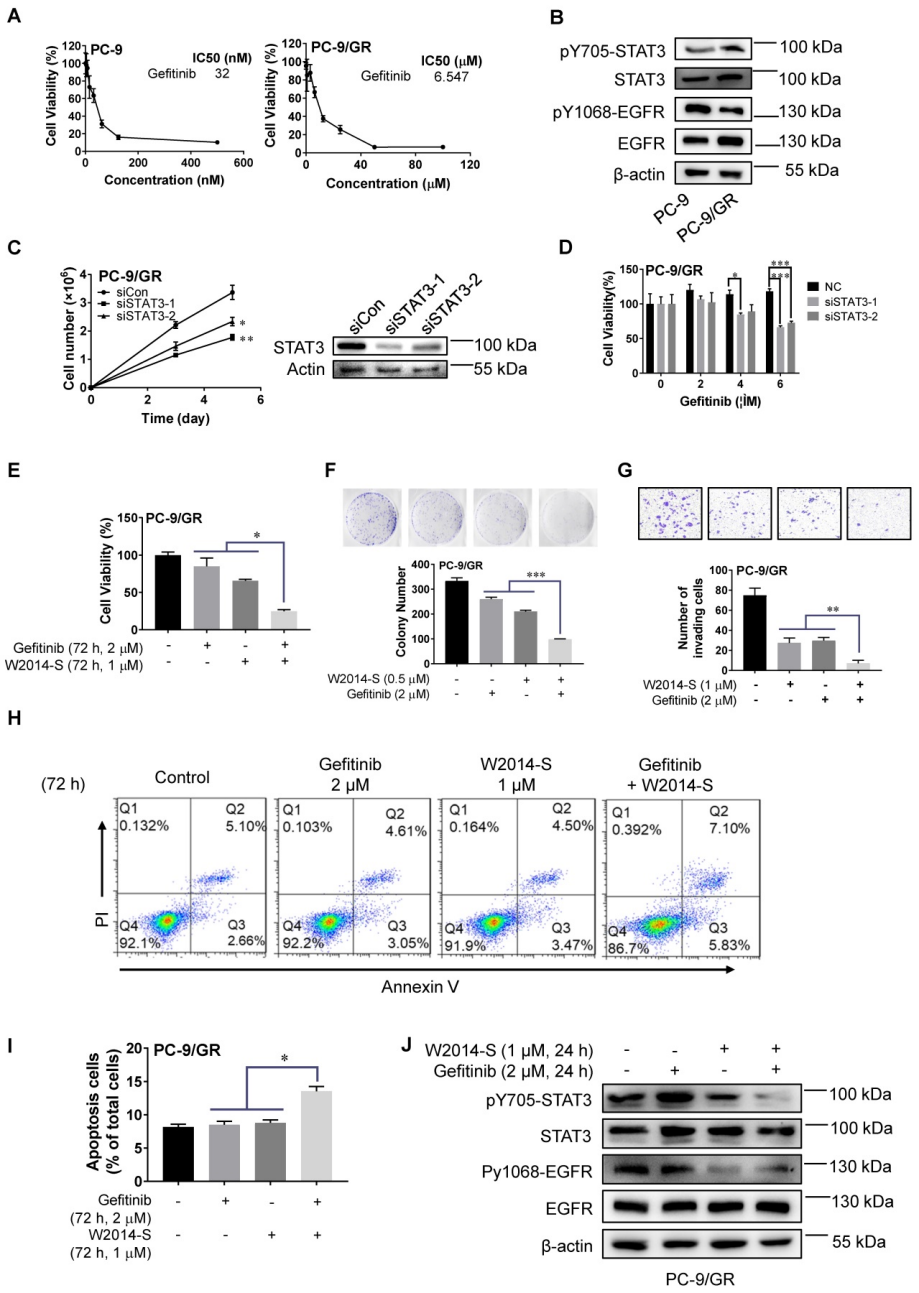
** W2014-S significantly enhanced gefitinib sensitivity* in vitro*. (A)** Cell proliferation and IC50 values of gefitinib at different concentrations for 72 h were measured in PC-9 and PC-9/GR cells. Each point represented the mean ± SEM, n=3. **(B)** The different expressions of pY705-STAT3, STAT3, pY1068-EGFR and EGFR in PC-9 and PC-9/GR cells were measured by western blot. **(C)** The silent effect of siRNA to STAT3 in PC-9/GR cells were detected by cell counting and western blot. Each point represented the mean ± SEM, n=3.^ *^P < 0.05, ^**^P < 0.01.** (D)** Cell viability of STAT3 knockdown was measured in PC-9/GR cells. Data were expressed as mean ± SEM, n=3. ^*^P < 0.05, ^**^P < 0.01, *^**^P < 0.001. **(E)** Cell proliferation of gefitinib (2 μM) and/or W2014-S (1 μM) for 72 h was measured in PC-9/GR cells. Data were expressed as mean ± SEM, n=3. ^*^P < 0.05.** (F)** Numbers of colonies of PC-9/GR cells treated with W2014-S (0.5 μM) and/or gefitinib (2 μM) were measured by colony formation assay. Data were expressed as mean ± SEM, n=3. ^*^P < 0.05, ^**^P < 0.01, *^**^P < 0.001. **(G)** Transwell cell invasion assay was used to detected cell invasion of PC-9/GR cells treated with W2014-S (1 μM) and/or gefitinib (2 μM) for 24 h. Data were expressed as mean ± SEM, n=3. ^*^P < 0.05, ^**^P < 0.01, *^**^P < 0.001. **(H-I)** PC-9/GR cells were treated with W2014-S (1 μM) and/or gefitinib (2 μM) for 72 h and apoptosis was measured by flow cytometry. PI means propidium iodide. The figures were modified due to the lower font size of labels in original picture. **(J)** The protein levels of pY705-STAT3, STAT3, pY1068-EGFR and EGFR in PC-9/GR cells treated with W2014-S (1 μM) and/or gefitinib (2 μM) for 24 h. Data were expressed as mean ± SEM, n=3. ^*^P < 0.05, ^**^P < 0.01, *^**^P < 0.001.

Previous studies suggested that over activated STAT3 may contribute to EGFR-TKI resistance. Here, we examine the role of STAT3 in TKI-resistance and choose gefitinib as a representative EGFR-TKI inhibitor in this study due to its wide application in lung cancer treatment. PC-9 cells harboring EGFR activating mutations were sensitive to gefitinib with an IC_50_ at 32 nM (Figure 5A). While PC-9 acquired resistance to gefitinib after continuous exposure to gefitinib at low concentration for a long time, and then it was termed as PC-9/GR with an IC_50_ at 6.547 μM (Figure 5A). The level of pY705-STAT3 and total-STAT3 was higher in PC-9/GR compared with PC-9 (Figure 5B). It suggested that increased level of STAT3 phosphorylation may contributed to the development of gefitinib resistance, and gefitinib could feedback-activate STAT3. While the expression of pY1068-EGFR and total-EGFR was not increased with gefitinib-resistance (Figure 5B). It revealed that over activated STAT3 alternative pathway contributes to gefitinib resistance in PC-9/GR. To further figure out the role of STAT3 in gefitinib resistance, PC-9/GR cells were transfected with siRNAs against STAT3. We found that knockdown of endogenous STAT3 significantly inhibited cell proliferation in PC-9/GR (Figure 5C). And PC-9/GR cells with silencing of STAT3 were more sensitive to gefitinib (Figure 5D). The above results suggested that the increased STAT3 activation in gefitinib-resistant cells contributes to the resistance of targeted therapies.

Next, we carried out an experiment to examine whether W2014-S, as a STAT3 inhibitor, can resensitize EGFR-TKI-resistant cells to gefitinib. As shown in Figure 5E and Figure S11, W2014-S at 1 μM was sufficient to resensitize PC-9/GR to gefitinib. Combination of W2014-S and gefitinib significantly improved the inhibitory effect compared with single treatment. Colony survival assay showed that the combination of W2014-S and gefitinib dramatically augmented the growth inhibitory effect compared with single treatment (Figure 5F). The activity of gefitinib on cancer cell invasion was also enhanced by combination with W2014-S in PC-9/GR in transwell cell invasion assay (Figure 5G). In addition, the level of pY705-STAT3 and pY1068-EGFR were both suppressed by the combination of gefitinib and W2014-S, although phosphorylation of STAT3 and EGFR was not inhibited by gefitinib only (Figure 5J). It suggested that W2014-S enhanced the sensitivity of resistant cells to gefitinib through inhibiting EGFR and STAT3 activities. Gefitinib treatment caused feedback activation of STAT3 in PC-9/GR, while W2014-S reduced the phosphorylation of STAT3. Gefitinib suppressed the activation of mitogen-activated protein kinase and induced cell apoptosis (43. To examine the synergistic effect of W2014-S and gefitinib on apoptosis, PC-9/GR cells were stained with PI and Annexin V-FITC and quantified by flow cytometry.

The results showed that W2014-S significantly enhanced gefitinib-induced apoptosis in PC-9/GR (Figure 5H-I). These results indicated that W2014-S significantly enhanced gefitinib sensitivity in PC-9/GR cells by inhibiting activated alternative STAT3 signaling pathway. We used erlotinib, anotherTKI inhibitors widely applied in clinic for NSCLC, to examine the effect of W2014-S. The level of pY705-STAT3 and pY1068-EGFR were both suppressed by the combination of gefitinib and W2014-S, stronger than single treatment. We found that W2014-S sensitized acquired resistance in PC-9/GR as shown in Figure S7B. The combination of W2014-S and erlotinib also showed significantly stronger inhibition than W2014-S or erlotinib alone in A549 and PC-9 cell line Figure S7C and S7D).

### W2014-S enhanced the anti-tumor effects of gefitinib in TKI-resistant lung cancer xenografts

**Figure 6:**
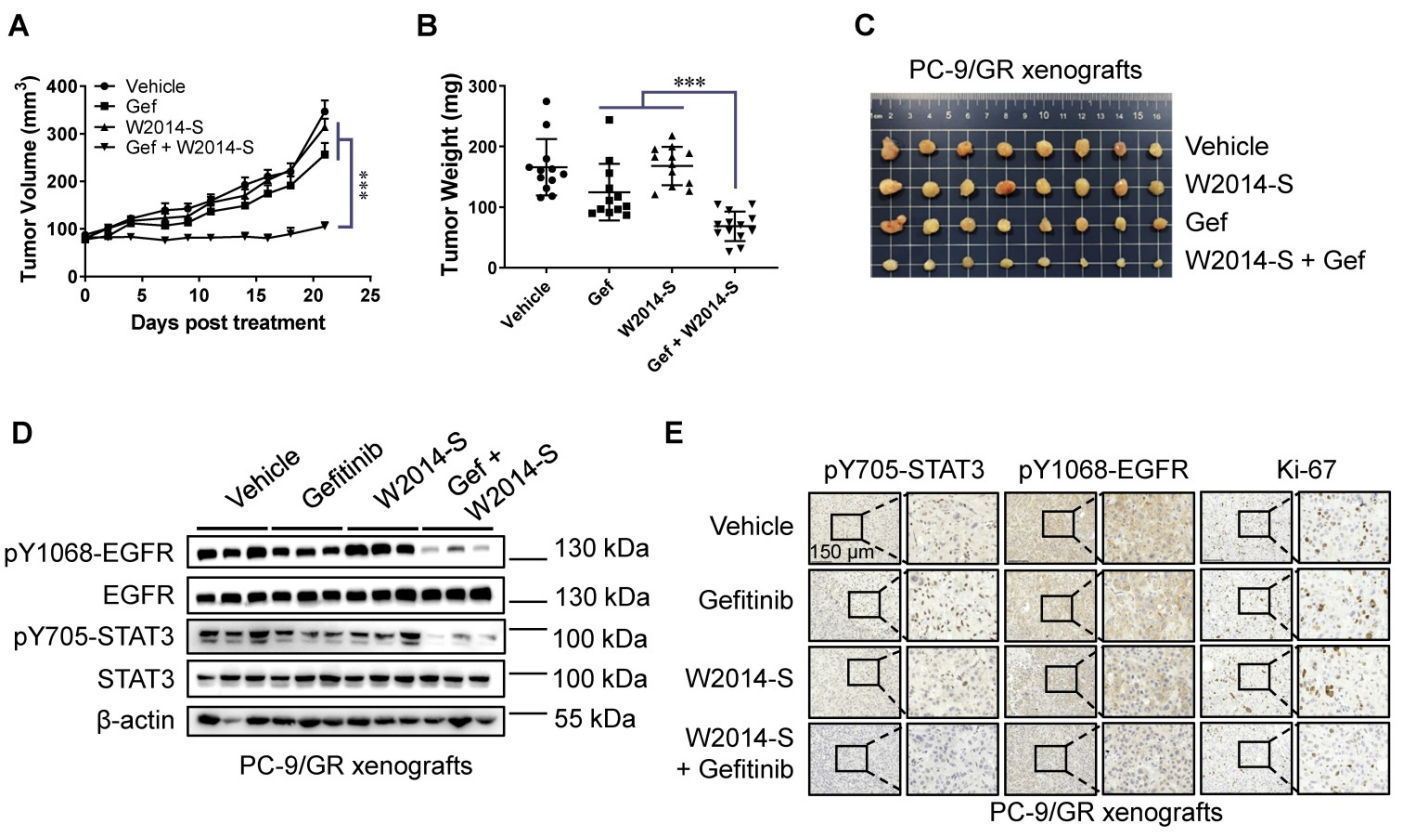
** Combination of W2014-S and gefitinib suppressed tumor growth.** Nu/nu mice were bearing PC-9/GR xenografts and treated with W2014-S (i.p. 10 mg/kg/d) and/or gefitinib (i.g. 50 mg/kg/d) for 21 d. (A) Tumor volume. Data were expressed as mean ± SEM, n=6. ^*^P < 0.05, ^**^P < 0.01, *^**^P < 0.001. (B) Tumor weight. Data were expressed as mean ± SEM, n=6. ^*^P < 0.05, ^**^P < 0.01, *^**^P < 0.001. (C) Tumor size and appearance. (D) The expressions of pY1068-EGFR, EGFR, pY705-STAT3 and STAT3 of PC-9/GR xenografts were detected by western blot. (E) IHC was used to detect the protein levels of pY705-STAT3, pY1068-EGFR and Ki-67 of PC-9/GR xenografts, and scale bar was 150 μm.

To further determine the therapeutic benefit of W2014-S in combination with gefitinib, we established a xenograft model of PC-9/GR cells in nude mice. Gefitinib showed a slight inhibitory effect on tumor growth of PC-9/GR by intragastric administration at a dose of 20 mg/kg per day. While co-administration of W2014-S at 10 mg/kg/day significantly suppressed xenograft tumor growth (Figure 6A-C). The tumor of PC-9/GR xenografts was weighted at end point. Combination group of W2014-S and gefitinib showed about 60% of tumor growth inhibition (TGI), much stronger than that of W2014-S or gefitinib alone (Figure 6B-C). Furthermore, expression of pY705-STAT3, STAT3, pY1068-EGFR and EGFR were examined by western blotting. The level of pY705-STAT3 and pY1068-EGFR was significantly suppressed in combination treatment group (Figure 6D). Immunohistochemical (IHC) staining also demonstrated that the level of pY705-STAT3, pY1068-EGFR and Ki-67 was inhibited by W2014-S and gefitinib combination (Figure 6E). There were no significant changes in body weights, or obvious signs of toxicity, such as loss of appetite, decreased activity, or lethargy during treatment (Figure S12. Taken together, W2014-S enhanced the anti-tumor effects of gefitinib in PC-9/GR xenografts through inhibition on alternatively activated STAT3 pathway. Downregulation of both pY705-STAT3 and pY1068-EGFR was only observed in combination treatment group. Combination of STAT3 inhibitor with gefitinib is more effective to inhibit TKI-resistant lung cancer xenograft growth than single treatment. To evaluate the acute toxicity of W2014-S, 40 mice were randomly distributed into 4 groups, and treated with W2014-S at 100mg/kg, 300mg/kg, and 500mg/kg by intraperitoneal injection. The mice were harvested and observed 14 days after treatment. We did observe no significant changes in body weights, or obvious signs of toxicity, such as loss of appetite, decreased activity, or lethargy during the observation. Furthermore, we examine the preliminary pharmacokinetic parameters for W2014-S in SD rats by intravenous and oral administration (Table 1 and Table S1-3 in Supplementary Information). A single dose of compound W2014-S at 3 mg/kg iv administration showed high volume of distribution at steady state (Vss) of 77.542 L/kg and moderate clearance (CL) of 205.8 mL min^-^1 kg^-^1, and long half-life (T1/2 = 13.6 h). Besides, a quick oral absorption (Tmax = 1.67 h), short half-life (T1/2 = 1.46 h) were at an oral dose of 10 mg/kg observed. While the oral bioavailability (F = 7.39%) needs to improve in the further modification.

## Discussion

It was reported that lung cancer patients with EGFR-TKI resistance cannot benefit from targeted therapy 13, 14. It is urgent to enhance the antitumor effect of targeted therapy for these patients. As a key regulator for multiple cellular processes including proliferation, differentiation, immune function and angiogenesis, STAT3 activation has been implicated as a critical mechanism in drug resistance for a range of oncogene driven cancers in targeted therapeutics. Activated STAT3 also plays an important role in immune response through regulation of immune checkpoint proteins and tumor environment cytokines 46, 47. Therefore, targeting STAT3 has a wide range of therapeutic implications. It will benefit lung cancer patients including the ones who are resistant to TKI treatment from multiple aspects.

To this end, we screened a series of small molecules with pharmacophore structure of imidazopyridine that was firstly utilized in STAT3 inhibitor discovery. W2014 was selected out from the in-house library. R and S enantiomers of W2014 showed similar affinity with STAT3 protein in SPR analysis, while they demonstrated distinct antitumor effects in following cell model studies. W2014-S and W2014-R were predicted to bind with different sites of STAT3 SH2 domain in molecular docking. The different binding pattern may contribute to their different anti-tumor activities. W2014-S strongly suppressed proliferation, survival, migration and invasion of lung cancer cells with aberrantly-active STAT3, and induced cell apoptosis. W2014-S selectively inhibited STAT3 activation without affecting upstream or other kinases, suggesting that its antitumor activity depends on STAT3 signaling inhibition. We further demonstrated that W2014-S can block direct STAT3-STAT3 dimerization in intact cells by co-immunoprecipitation study. The accumulation of pY705-STAT3 in the nucleus was downregulated by W2014-S as shown in confocal imaging. Luciferase reporter studies showed that W2014-S inhibited STAT3-dependent transcriptional activity dose-dependently. Moreover, W2014-S significantly inhibited growth of subcutaneous mouse xenografts of NSCLC cell line and PDX harboring aberrantly active STAT3. The level of pY705-STAT3 in tumor tissue was significantly inhibited by W2014-S in western blotting and IHC analysis. Moreover, the expression of STAT3 downstream genes, including c-Myc, Bcl-2 and Bcl-xL in tumor tissue were also downregulated by W2014-S. Thus, W2014-S effectively inhibits STAT3 signaling with potent anti-tumor activities *in vitro* and* in vivo*. Although we demonstrated that W2014-S selectively inhibit STAT3 signaling and inhibit tumor growth *in vitro* and *in vivo*, without co-crystal information, it is hard to tell where and how this compound interact with STAT3. Further structure biology study will reveal more information for detailed mechanism.

Previous studies showed that high level of STAT3 phosphorylation in lung cancer patients may contribute to EGFR-TKIs acquired resistance 19, 21, 22, 48. Therefore, we further investigated the effect of W2014-S in TKI-resistance. We used PC-9/GR, a NSCLC cell line with acquired resistance to gefitinib by continuous exposure to gefitinib, to examine the role of STAT3 and effect of W2014-S. The protein levels of pSTAT3 and total-STAT3 were found higher in PC-9/GR than those in PC-9. Knockdown of endogenous STAT3 significantly inhibited cell proliferation and resensitized PC-9/GR to gefitinib treatment. These data suggested that alternatively activated STAT3 signaling pathways contributed to TKI resistance in PC-9/GR. We further demonstrated that W2014-S resensitized EGFR-TKI-resistant NSCLC to gefitinib in cell proliferation, colony survival assay and transwell cell invasion study. Furthermore, W2014-S and gefitinib synergistically induced cell apoptosis in PC-9/GR. In addition, the protein levels of pY705-STAT3 and pY1068-EGFR were both suppressed by combination of gefitinib and W2014-S, but not by gefitinib alone. It suggested that W2014-S may enhance the sensitivity of resistant cells to gefitinib through inhibition on EGFR and STAT3 activation. Gefitinib treatment may cause feedback activation of STAT3 in PC-9/GR, while W2014-S reduced the phosphorylation of STAT3. To further determine the therapeutic benefit of W2014-S in combination with gefitinib, we established the xenograft model of PC-9/GR cells. Gefitinib showed a slight inhibitory effect on tumor growth, while combination of W2014-S and gefitinib significantly suppressed xenograft tumor growth. Further immunohistochemical staining and western blotting showed that pY705-STAT3 and pY1068-EGFR were reduced by combination treatment. We demonstrated that combination EGFR blockade and STAT3 inhibition was more effective in inhibiting TKI resistant lung cancer cell xenograft growth than inhibition of either pathway alone.

However, molecular mechanisms underlying acquired TKI resistance is involved in various pathways and much more complicated than we expected. The cross talking between EGFR signaling and STAT3 pathway is also complex. STAT3 may work as a downstream of EGFR signaling pathway and EGFR can activate STAT3 pathway from several aspects. It may trigger the phosphorylation of STAT3, or upregulate expression of IL-6 to promote STAT3 activation, or mediate STAT3 signaling pathway through Raf-MEK-ERK signaling axis 49-51. It was reported that nuclear EGFR interact with STAT3 to regulate expression of target genes 49, 51. Overall, the upregulation of STAT3 via a positive feedback loop is a primary mechanism of TKI resistance in targeted therapy. We guess that the interruption on cross talking between STAT3 and EGFR by W2014-S mainly depends on its function as a STAT3 inhibition, but the detailed mechanism need to be explored in future study with more solid evidences. The crosstalking with other pathways including IL-37, HIF-1 alpha and S1PR1 also contribute to drug sensitivity in cancer therapeutics 52, 53. Furthermore, STAT3 can be activated by both canonical and noncanonical signaling pathway, and mediated by post-translational modification. Therefore, the role of mitochondria STAT3 in drug resistance and signaling cross talking cannot be neglected. AL Wong and Boon-Cher Goh *et al* found that the upregulation of oxidative phosphorylation is also an important mechanism for drug resistance of targeted therapy 54-56. Function of mitochondria STAT3 may provide link between cancer cell metabolism and drug resistance. Moreover, OPB compounds (STAT3 inhibitors in clinical study) was found to inhibit mitochondria function via interaction with mitochondria STAT3 42, 54, 56, 57. Therefore, combination of OXPHOS inhibition and TKI may provide a novel therapeutic strategy to overcome resistance 9, 56, 58. However, the mitochondria dysfunction by targetting mitochondria STAT3 may link to toxicity profiles of STAT3 inhibitors. Nevertheless, targeting cancer cell metabolism is an attractive stragy worthy to explore deeply.

Taken together, in the present study we demonstrated that W2014-S, as a novel STAT3 inhibitor, effectively inhibited STAT3 signaling with potent anti-tumor activities in human lung cancer, and enhanced the sensitivity of resistant NSCLC to gefitinib *in vitro* and *in vivo*. This provided a potential combination therapeutic strategy for the treatment of EGFR-TKI resistance lung cancer.

## Supplementary Material

Supplementary figures and tables.

## Figures and Tables

**Table 1:** Preliminary Pharmacokinetic Parameters for W2014S ^a^

Parameter	3 mg/kg *iv*	10 mg/kg *po*
CL (mL min^-1^kg^-1^)	205.8	
V_ss_ (L/kg)	77.542	
T_1/2_ (h)	13.6	1.46
T_max_ (h)		1.67
C_max_ (ng/mL)		23.8
AUC_0-last_ (ng•h/mL)	222	54.6
AUC_0-inf_ (ng•h/mL)	244	56.1
F (%)		7.39

^a^Values are the average of three runs. Vehicle: DMSO/0.5% HPMC (5/95, v/v/). CL, clearance; V_ss_, volume of distribution; T_1/2_, half-life; T_max_, time of maximum concentration; C_max_, maximum concentration; AUC, area under the plasma concentration-time curve; F, oral bioavailability.

## Abbreviations

STAT3: Signal Transducer and Activator of Transcription 3
EGFR: Epidermal growth factor receptor
TKI: Tyrosine kinase inhibitor
NSCLC: Non-small cell lung cancer
PFS: Progression-free-survival
PDX: Patient-derived tumor xenograft
SPR: Surface plasmon resonance
SH2: Src-homology 2
TGI: Tumor growth inhibition
