# Supplementary material for: A novel STAT3 inhibitor W2014-S regresses human non-small cell lung cancer xenografts and sensitizes EGFR-TKI acquired resistance
Source: Theranostics. 2021 Jan 1;11(2):824–40. doi: 10.7150/thno.49600 (PMC7738869; doi:10.7150/thno.49600)
Supplement: Supplementary file 1 — Supplementary figures and tables. [file thnov11p0824s1.pdf]

Supplementary Information

Table S1-3. Raw data for Preliminary Pharmacokinetic studies

Table S1. Concentrations (ng/mL) of W2014S in rat plasma after p.o. administration of W2014S 10 mg/kg

Time (h)	1	2	3	Mean	SD
0.25	7.10	21.9	29.9	19.6	11.6
0.50	10.0	27.4	30.0	22.5	10.9
1.00	8.00	18.7	21.7	16.1	7.2
2.00	5.70	9.40	6.60	7.23	1.93
4.00	13.9	4.80	4.80	7.83	5.25
8.00	1.20	0.400	0.300	0.633	0.493
24.0	BLQ	BLQ	BLQ	0.00	-

BLQ: Below LLOQ (LLOQ = 0.3 ng/mL)

Table S2. Concentrations (ng/mL) of W2014S in rat plasma after i.v. administration of W2014S 3 mg/kg

Time (h)	4	5	6	Mean	SD
0.05	454	335	339	376	68
0.25	159	145	134	146	13
0.75	59.3	66.9	80.1	68.8	10.5
2.00	13.4	17.6	22.6	17.9	4.6
4.00	2.90	3.60	5.00	3.83	1.07
8.00	1.10	2.60	2.20	1.97	0.78
24.0	0.900	1.30	1.20	1.13	0.21

BLQ: Below LLOQ (LLOQ = 0.3 ng/mL)

Table S3. Pharmacokinetics parameters of W2014S after p.o and i.v. administration in rat

	Animal	$T_{1/2}$	T_{max}	C_{max}	AUC_{last}	AUC_{INF_obs}	$CL_{\_obs}$	MRT_{INF_obs}	$Vss_{\_obs}$	F
		(h)	(h)	ng/mL	h*ng/mL	h*ng/mL	mL/min/kg	h	mL/kg	(%)
p.o.	1	1.88	4.00	13.9	54.6	57.9	-	3.89	-	7.39
	2	1.29	0.50	27.4	54.6	55.3	-	2.08	-	
	3	1.21	0.50	30.0	54.5	55.0	-	1.87	-	
	mean	1.46	1.67	23.8	54.6	56.1	-	2.61	-	
	SD	0.36	2.02	8.6	0.1	1.6	-	1.11	-	
i.v.	4	15.3	-	-	208	228	219	5.79	76143	
	5	14.2	-	-	220	247	202	7.48	90763	
	6	11.2	-	-	236	255	196	5.59	65720	
	mean	13.6	-	-	222	244	205.8	6.3	77542	
	SD	2.1	-	-	14	14	11.9	1.0	12580	

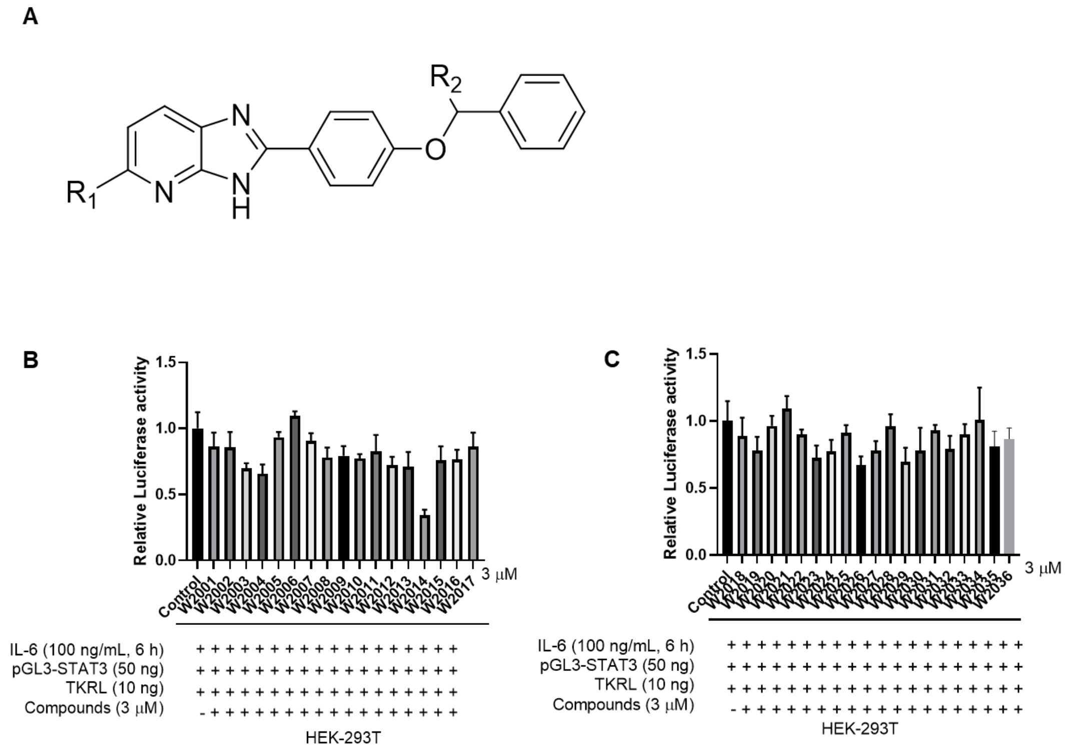

Figure S1. Primary screening of compounds through STAT3-depdent luciferase reporter assay. A) Core chemical structure for W2014 series compounds, B) STAT3-depdent luciferase reporter assay for compound W2001-W2017; B) STAT3-depdent luciferase reporter assay for compound W2018-W2036.

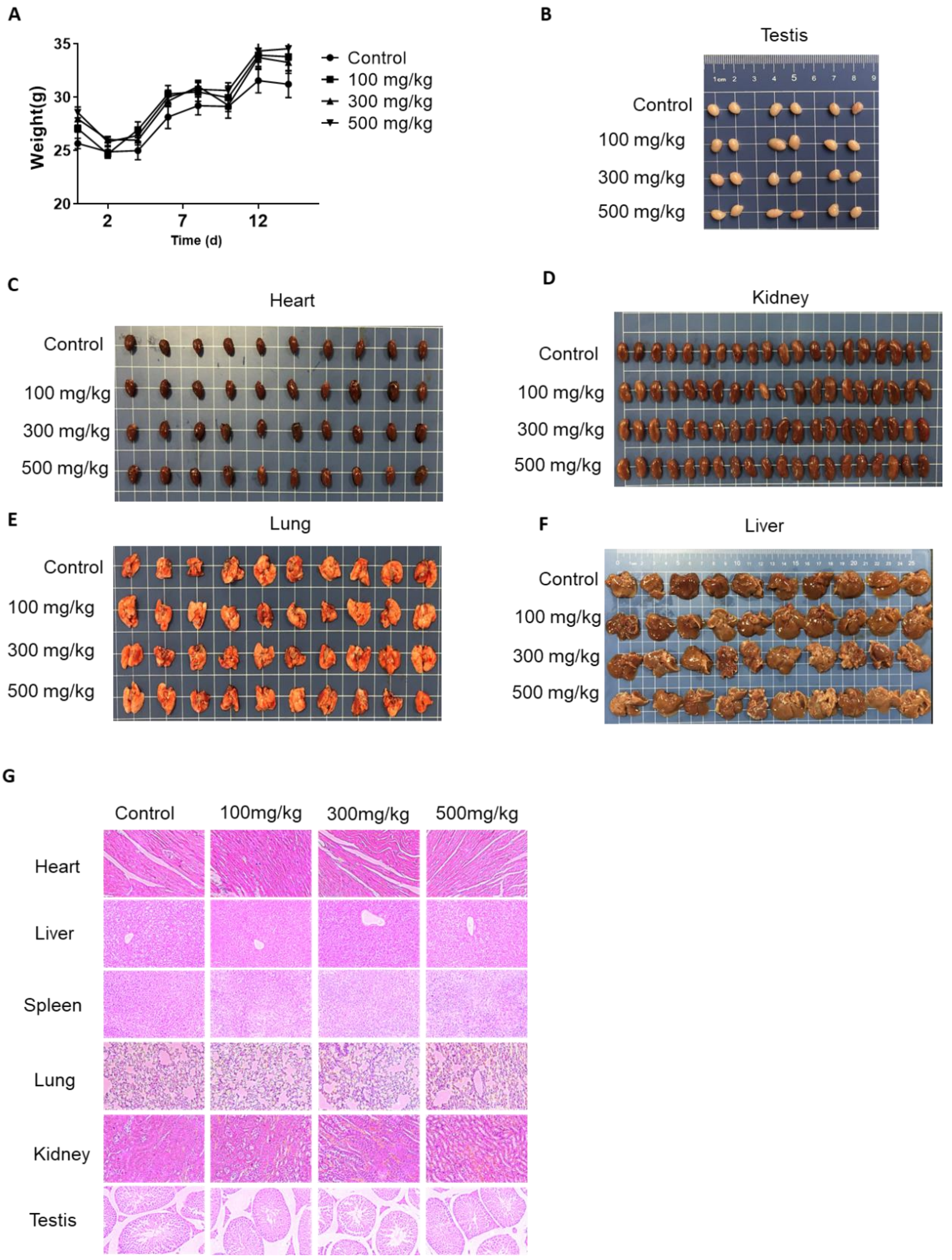

Figure S2. Acute toxicity study for W2014-S.

A) Body weight curve of mice after treatment for 14 days; B) The size and appearance of testis; C) The size and appearance of heart; D) The size and appearance of kidney; E) The size and appearance of lung; F) The size and appearance of liver; G) HE staining for heart, liver, spleen, lung, kidney, and testis from each group.

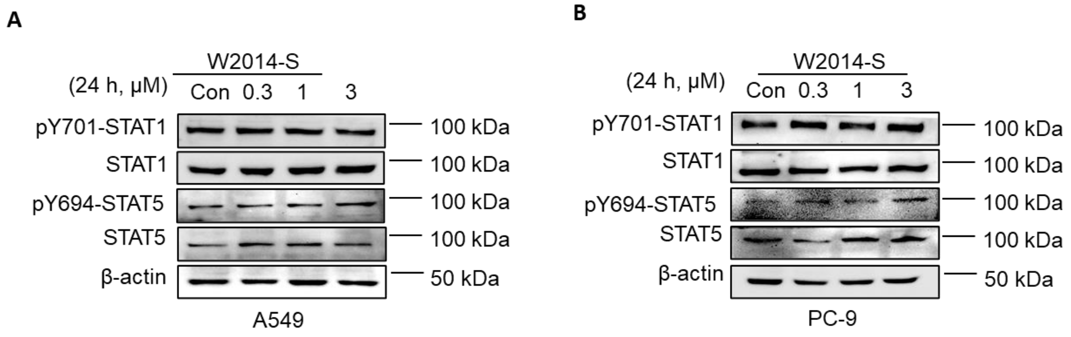

Figure S3. Effect of W2014S on STAT1 and STAT5 activation in A549 and PC-9 cell line. A) Effect of W2014-S on STAT1 and STAT4 phosphorylation in A549 cell line. B) Effect of W2014-S on STAT1 and STAT5 phosphorylation in PC-9 cell line.

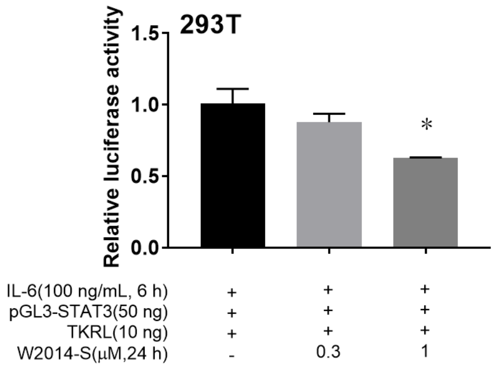

Figure S4. Dual-luciferase reporter assay was used to measure the translational activity of 293T cells treated with W2014-S at 0, 0.3, 1 μM for 24 h. Data were expressed as mean ± SEM, n=3. *P < 0.05.

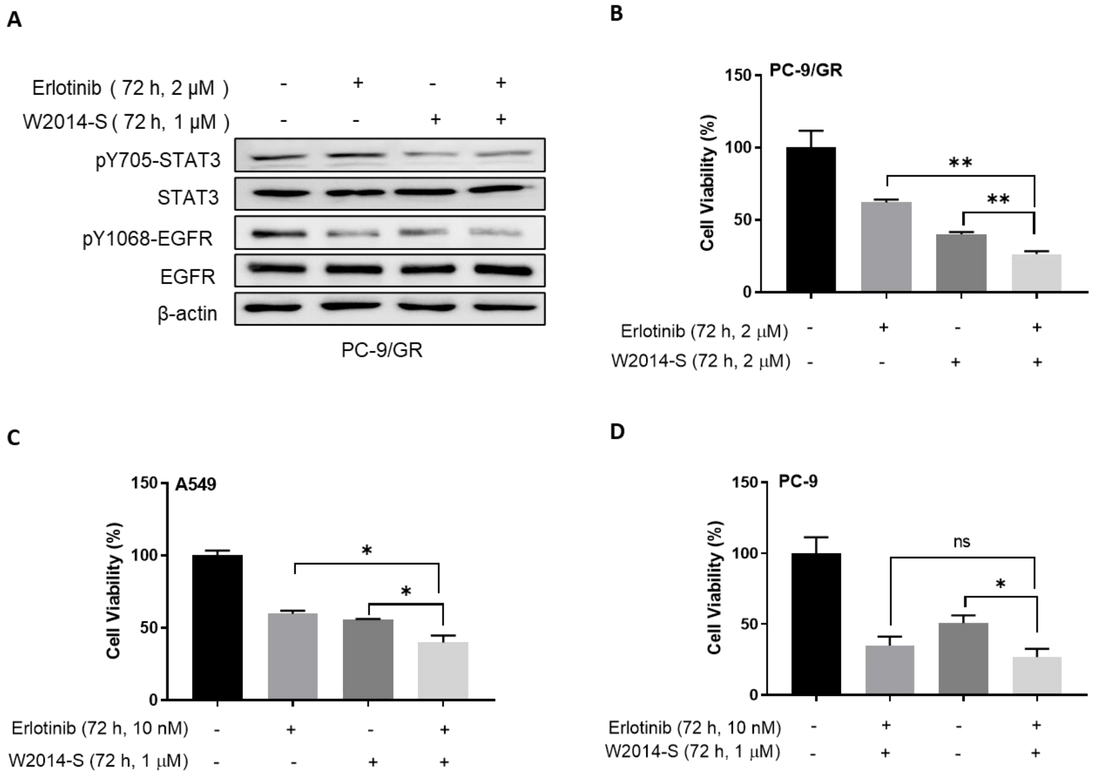

Figure S5. W2014-S enhanced erlotinib sensitivity in vitro. A) The protein levels of pY705-STAT3, STAT3, pY1068-EGFR and EGFR in PC-9/GR cells treated with W2014-S (1 μM) and/or erlotinib (2 μM) for 24 h. B) PC-9/GR cells were treated with W2014-S and erlotinib alone or combination for 72h and performed proliferation assay. C) A549 cells were treated with W2014-S and erlotinib alone or combination for 72h and performed proliferation assay. D) PC-9 cells were treated with W2014-S and erlotinib alone or combination for 72h and performed proliferation assay. Data were expressed as mean ± SEM, n=3. *P < 0.05, **P < 0.01.

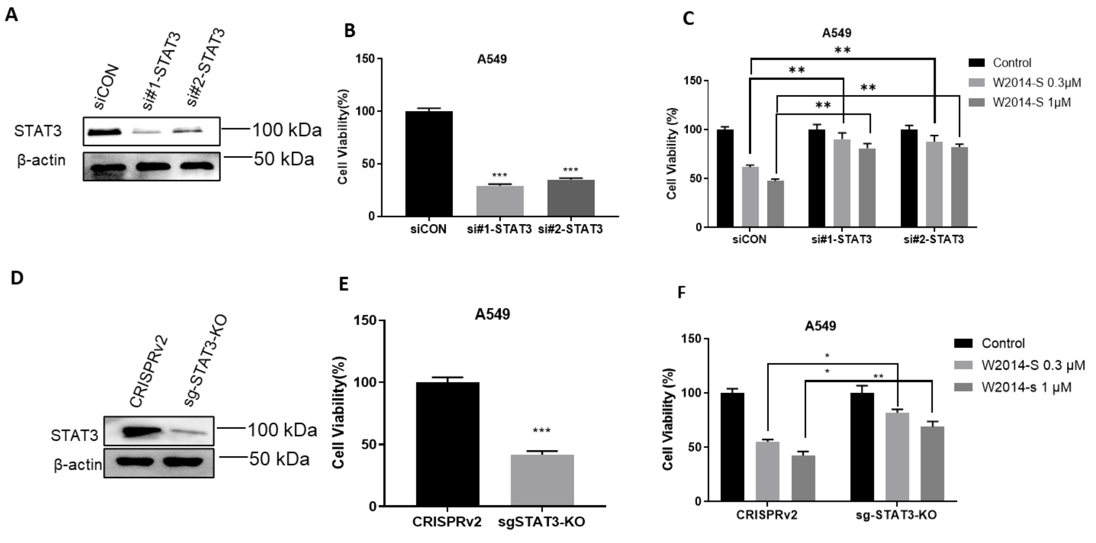

Figure S6. Deletion of STAT3 significantly attenuated anti-cancer potency of W2014-S. A) Western blotting was performed to examine whether STAT3 was knocked by siRNA interference; B) Cell proliferation was measured to evaluate whether knock down of STAT3 inhibit cell viability; C) Cell proliferation assay was measured to examine whether anti-cancer activity of W2014-S was attenuated by STAT3 knock down; D) Western blotting was performed to examine whether STAT3 was knocked by Crispr/Cas9; E) Cell proliferation was measured to evaluate whether knock out of STAT3 inhibit cell viability; F) Cell proliferation assay was measured to examine whether anti-cancer activity of W2014-S was attenuated by STAT3 knock out. Data were expressed as mean ± SEM, n=3. *P < 0.05, **P < 0.01.

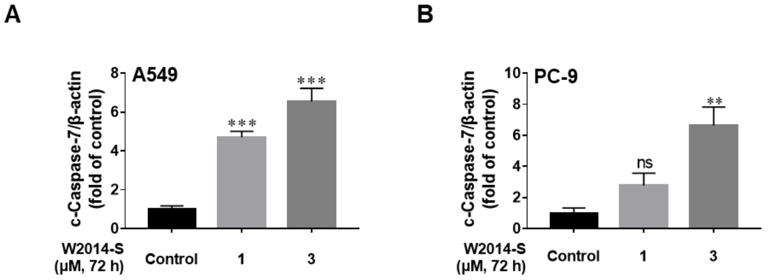

Figure S7. Quantification of western blot. A) Quantification for c-Caspase-7/Actin in A549 cells in Figure2J; B) Quantification for c-Caspase-7/Actin in PC9 cells in Figure2J.

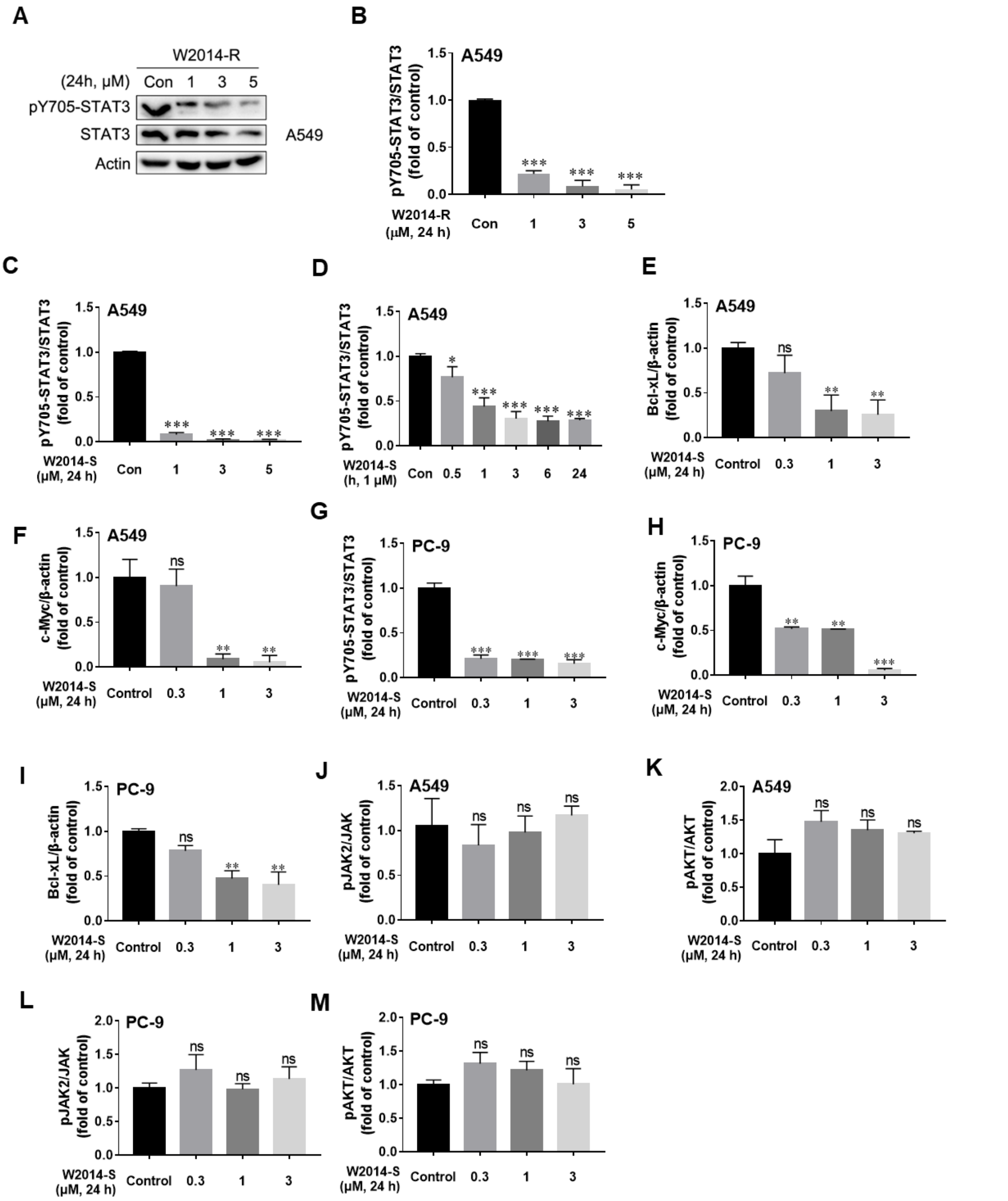

Figure S8. Quantification of western blot. A) Western blot was used to detect the expression of STAT3 and pY705-STAT3 in A549 cells treated with W2014-R at 0, 1, 3 and 5 μM for 24 h; B) Quantification for pY705-STAT3/Actin in A549 cells in Figure S8A; C) Quantification for pY705-STAT3/Actin in A549 cells in Figure 3A; D) Quantification for pY705-STAT3/Actin in A549 cells in Figure 3B; E) Quantification for Bcl-xL/Actin in A549 cells in Figure 3C; F) Quantification for c-Myc/Actin in A549 cells in Figure 3C; G) Quantification for pY705-STAT3/Actin in PC9 cells in Figure 3C; H) Quantification for c-Myc/Actin in PC9 cells in Figure 3C; I) Quantification for Bcl-xL /Actin in PC9 cells in Figure 3C; J) Quantification for pJAK/JAK in A549 cells in Figure 3D;

K) Quantification for pAKT/AKT in A549 cells in Figure 3D; L) Quantification for pJAK/JAK in PC9 cells in Figure 3D; M) Quantification for pAKT/AKT in A549 cells in Figure 3D.

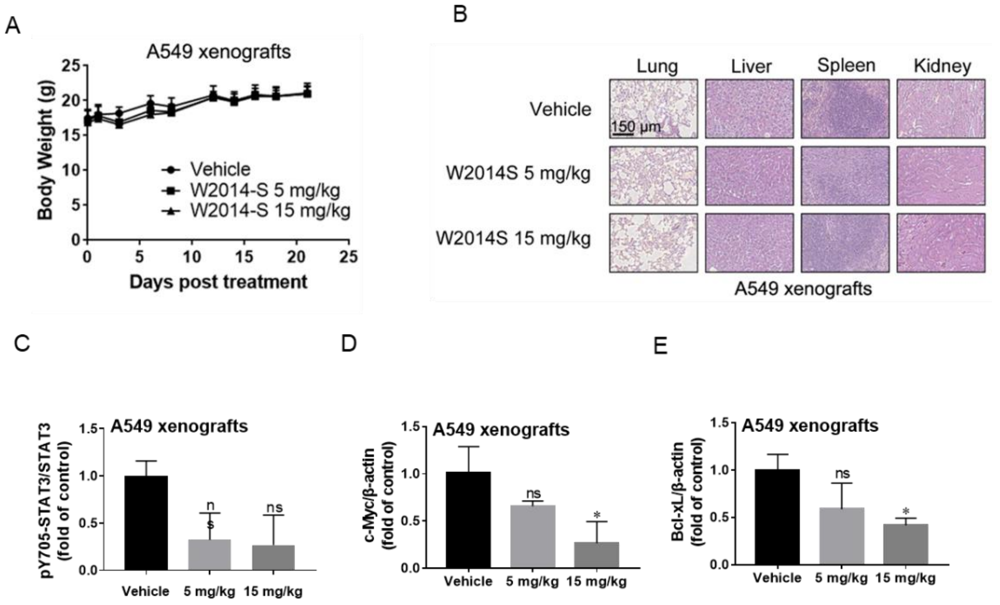

Figure S9. Toxicity evaluation of W2014-S in mice of A549 xenografts model. A) Plots of body weight during days of treatment for mice. Mice were bearing A549 xenografts and treated with W2014-S via *i.v.* 5 or 15 mg/kg or vehicle every day; B) HE staining of lung, liver, spleen and kidney of each group in A549 xenografts model; C) Quantification for pY705-STAT3/STAT3 in Figure 4G; D) Quantification for c-Myc/β-actin in Figure 4G; E) Quantification for Bcl-xL/β-actin in Figure 4G. All data were expressed as the mean ± SEM. n=6, *$P < 0.05$, **$P < 0.01$, compared with vehicle group.

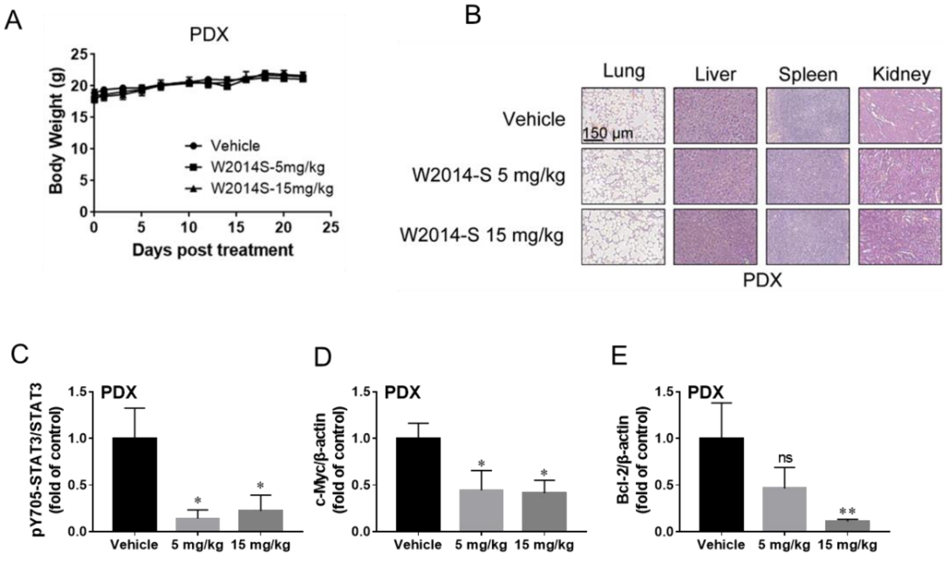

Figure S10. Toxicity evaluation of W2014-S in mice of lung PDX model. A) Body weights of mice; B) HE staining of lung, liver, spleen and kidney of each group in PDX model and scale bar was 150 μm; C) Quantification for pY705-STAT3/STAT3 in Figure 4H; D) Quantification for c-Myc/β-actin in Figure 4H; E) Quantification for Bcl-xL/β-actin in Figure 4H. All data were expressed as the mean ± SEM. n=6, *P < 0.05, **P < 0.01, compared with vehicle group.

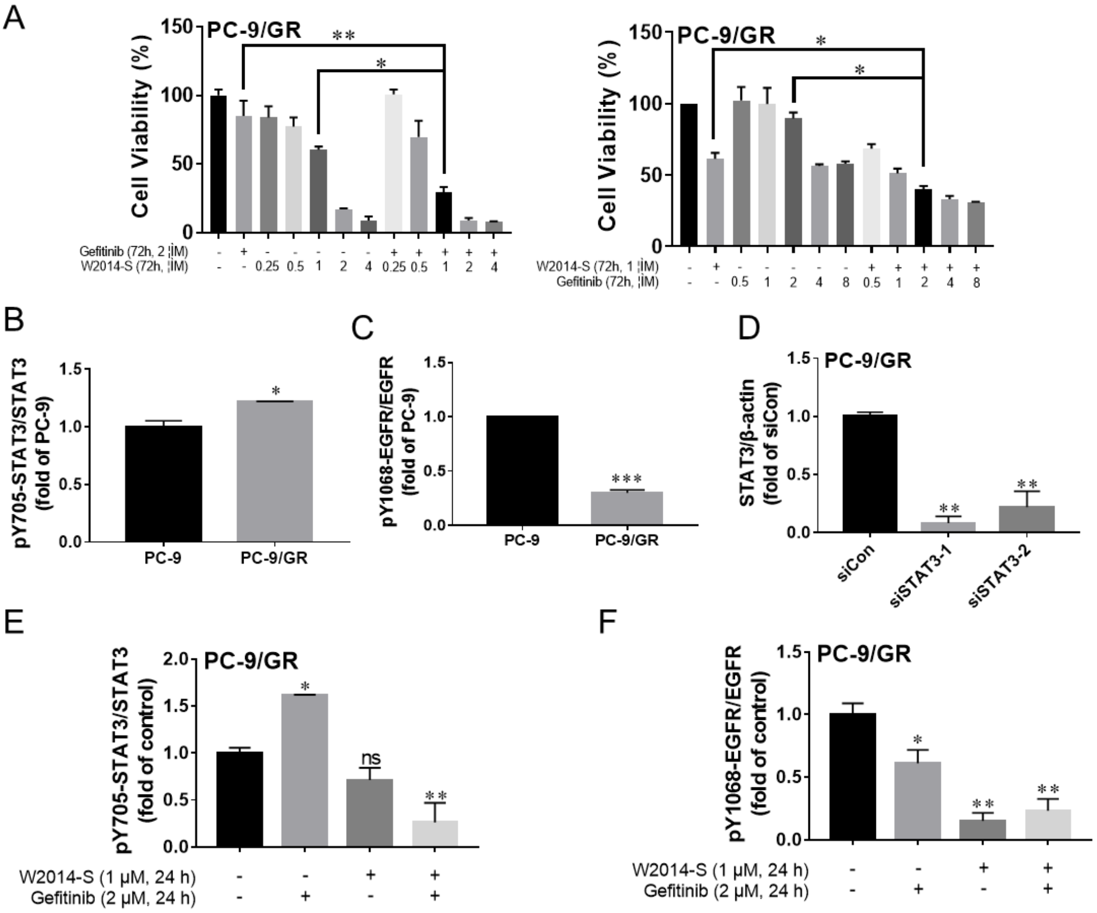

Figure S11. W2014-S enhanced the sensitivity of PC-9/GR cells to gefitinib treatment. A）PC-9/GR cells were treated with W2014-S in the absence or presence of gefitinib for 72h and PC-9/GR cells were treated with gefitinib in the absence or presence of W2014-S for 72h; B) Quantification for pY705-STAT3/STAT3 in Figure 5B; C) Quantification for p1068-EGFR/EGFR in Figure 5B; D) Quantification for STAT3/β-actin in Figure 5C; E) Quantification for pY705-STAT3/STAT3 in Figure 5J; F) Quantification for p1068-EGFR/EGFR in Figure 5J. All data shown are expressed as the mean ± SEM, *P < 0.05, **P < 0.01.

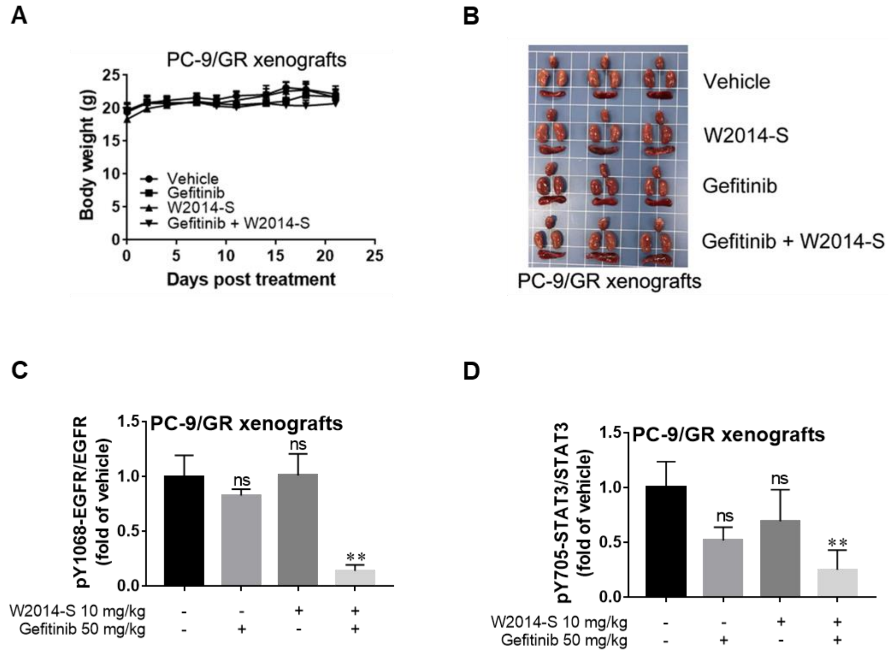

Figure S12. Toxicity evaluation of combination of W2014-S and gefitinib in PC-9/GR xenografts model. A) Body weights of mice. Data were expressed as mean ± SEM, n=6. *P < 0.05, **P < 0.01, ***P < 0.001. B）The size and appearance of heart, spleen and kidney of each group in PC-9/GR xenografts model. C）Quantification for p1068-EGFR/EGFR in Figure 6D. D) Quantification for pY705-STAT3/STAT3 in Figure 6D.

Chemical methods and synthetic route

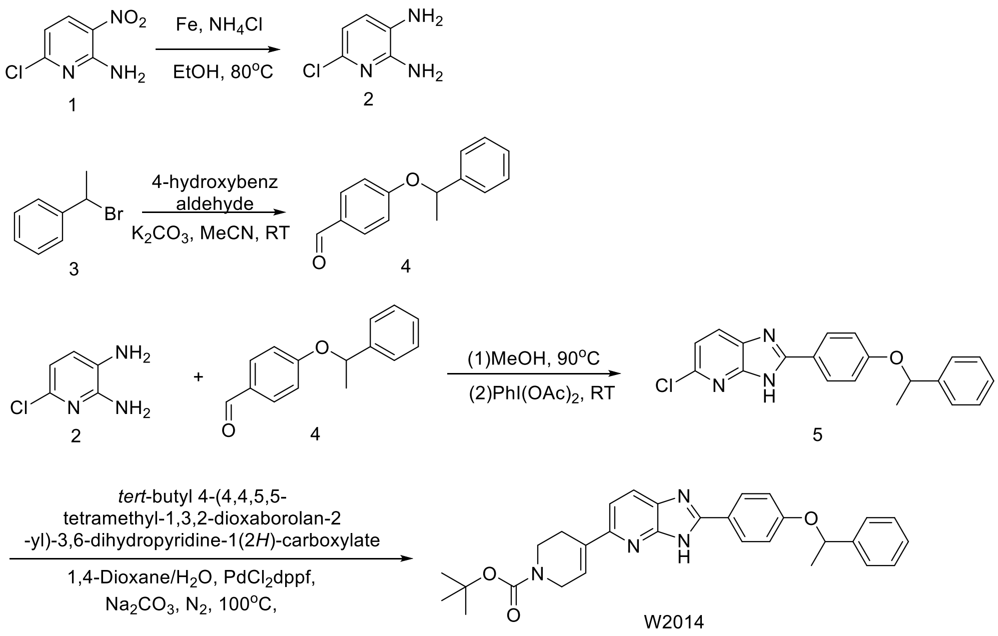

Figure S13. Chemical synthetic route for W2014

Step 1： Compound 1 (1 eq), iron powder (3 eq) and ammonium chloride (4 eq) were added into 20 ml of ethanol. The rection mixture was heated to relux for 2 h. Then the mixture was filtered through celite and the filtrate was condensed under vacuum to afford the crude product, which was purified by column chromatography (PE/EA = 3 : 1) to afford compound 2 with the yield of 78%.^{1}H NMR (500 MHz, $CDCl_3$) δ 6.85 (d, J = 7.8 Hz, 1H), 6.60 (d, J = 7.8 Hz, 1H), 4.45 (s, 2H), 3.28 (s, 2H). ESI-MS: 145.0 $[M+H]^+$

Step 2: Compound 3, 4-hydroxybenzaldehyde (1.1 eq), carbon potassium acid (2 eq) were dissolved in 40 mL acetonitrile, and stirred overnight at room temperature. The reaction mixture was filtered and the filtrate was condensed under vacuum to afford the crude product, which was purified by colomn chromatography (PE/EA = 20 : 1) to afford compound 4 with the yield of 90%.^{1}H NMR (400 MHz, $CDCl_3$) δ 9.84 (s, 1H), 7.80 – 7.73 (m, 2H), 7.41 – 7.34 (m, 4H), 7.32 – 7.28 (m, 1H), 6.99 (d, J = 8.7 Hz, 2H), 5.44 (q, J = 6.4 Hz, 1H), 1.70 (d, J = 6.4 Hz, 3H). ESI-MS: 227.2 $[M+H]^+$

Step3: Compound 2 (1 eq) and compound 4 (1.1 eq) were dissolved in 10 mL methanol. The reaction solution was heated at 90 ℃ for 30 min. Then $PhI(OAc)_2$ (1 eq) was added to above solution at room temperature and .stirred overnight at room temperature. The reaction solution was filtered through celite and the filtrate was condensed under vacuum to afford the crude product, which was purified by column chromatography (PE/EA = 5 : 1) to afford compound 5 with the yield of 53%.^{1}H NMR (400 MHz, $CDCl_3$) δ 11.90 (s, 1H), 8.05 – 7.70 (m, 3H), 7.36 (d, J = 7.6 Hz, 4H), 7.28 (s, 1H), 7.05 (s, 1H), 6.87 (t, J = 40.5 Hz, 2H), 5.39 (d, J = 6.1 Hz, 1H), 1.68 (d, J = 5.8 Hz, 3H). ESI-MS: 350.8 $[M+H]^+$

Step4: Compound 5 (1 eq), tert-butyl 4-(4,4,5,5-tetramethyl-1,3,2-dioxaborolan-2-yl)-3,6-dihydropyridine-1(2H)-carboxylate (2 eq), $PdCl_2$dppf (0.1 eq) and sodium carbonate (2 eq) were added in a mixture of 12 mL 1,4-dioxane and 4 mL of water under nitrogen atmosphere and the mixture was heated to 100 ℃ for 8 h. The reaction solution was filtered through celite and the filtrate was condensed under vacuum to afford the crude product, which was purified by column chromatography (PE/EA = 3 : 1) to afford W2014 with the yield of 71%.^{1}H NMR (400 MHz, DMSO-d_6) δ 13.34 (s, 1H), 12.89 (s, 1H), 8.08 (d, J = 8.2 Hz, 3H), 7.94 (d, J = 8.1 Hz, 1H), 7.45 (d, J = 7.6 Hz, 4H), 7.36 (t, J

= 7.5 Hz, 3H), 7.27 (t, J = 7.2 Hz, 1H), 7.07 (d, J = 7.9 Hz, 3H), 6.64 (s, 1H), 5.64 (d, J = 6.3 Hz, 1H), 4.07 (s, 3H), 3.57 (t, J = 5.3 Hz, 3H), 2.65 (s, 3H), 1.59 (d, J = 6.2 Hz, 4H), 1.44 (s, 9H); ^{13}C NMR (126 MHz, DMSO-d_6) δ 159.72, 154.43, 153.08, 151.16, 149.34, 143.09, 135.48, 135.32, 129.04, 128.55, 128.01, 126.40, 126.19, 122.61, 116.59, 114.36, 79.32, 75.37, 28.59, 26.24, 24.55;ESI-MS: 497.0 $[M+H]^+$; HRMS(ESI-TOF): *m/z* calculated for $C_{30}H_{32}N_4O_3$ $[M+H]^+$: 497.2547, found: 497.2563.

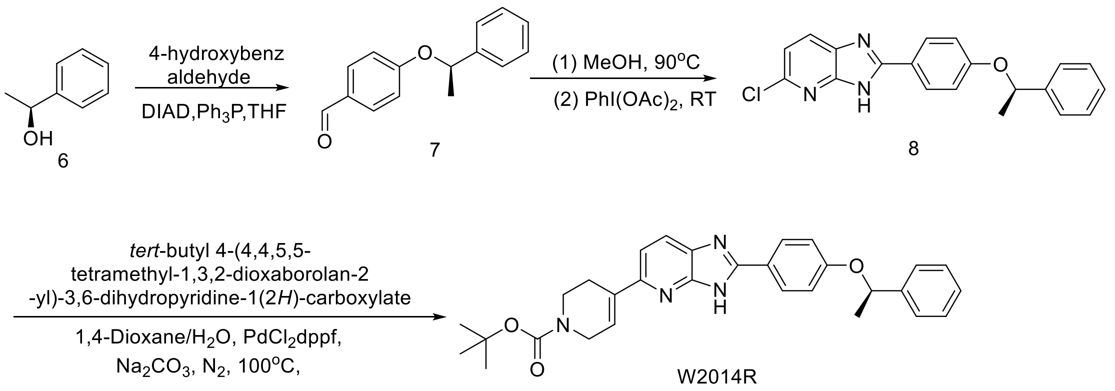

Figure S14. Chemical synthetic route for W2014-R

Step1: Triphenylphosphine (1.8 eq) was dissolved in 80 mL tetrahydrofuran and cooled at ice bath. DIAD (1.8 eq) was added into the solution drop wisely. After 20 min, the solution of compound 6 (1 eq) and 4-hydroxybenzaldehyde (1 eq) in 20 ml tetrahydrofuran was added into above reaction solution and was stirred at room temperature for 10 h. The reaction solution was concentrated under vacuum and then purified by chromatography (PE/EA = 5: 1) to afford compound 8 in a yield of 44%. ^{1}H NMR (400 MHz, $CDCl_3$) δ 9.84 (s, 1H), 7.78 – 7.74 (m, 2H), 7.39 – 7.36 (m, 4H), 7.32 – 7.28 (m, 1H), 6.99 (d, J = 8.7 Hz, 2H), 5.44 (q, J = 6.4 Hz, 1H), 1.70 (d, J = 6.4 Hz, 3H). ESI-MS: 227.2 $[M+H]^+$

Step 2: Following a similar procedure as that for preparation of compound 5 afforded compounds 8 from compound 7 with a yield of 58%.^{1}H NMR (400 MHz, $CDCl_3$) δ 11.90 (s, 1H), 8.05 – 7.69 (m, 3H), 7.35 (d, J = 7.6 Hz, 4H), 7.28 (s, 1H), 7.06 (s, 1H), 6.87 (t, J = 40.5 Hz, 2H), 5.39 (d, J = 6.1 Hz, 1H), 1.67 (d, J = 5.8 Hz, 3H). ESI-MS: 350.8$[M+H]^+$

Step 3: Following a similar procedure as that for preparation of W2014 afforded W2014-R from compound 8 with a yield of 67%. ^{1}H NMR (500 MHz, DMSO) δ 8.07 (d, J

= 7.8 Hz, 2H), 7.92 (s, 1H), 7.44 (d, *J* = 7.4 Hz, 3H), 7.36 (t, *J* = 7.1 Hz, 2H), 7.27 (t, *J* = 7.0 Hz, 1H), 7.07 (d, *J* = 6.1 Hz, 2H), 5.63 (d, *J* = 5.8 Hz, 1H), 4.07 (s, 2H), 3.57 (s, 2H), 2.65 (s, 2H), 1.59 (d, *J* = 5.7 Hz, 3H), 1.44 (s, 9H); ^{13}C NMR (126 MHz, DMSO) δ 159.75, 154.44, 143.08, 129.04, 128.59, 128.00, 126.18, 122.61, 116.59, 114.35, 79.31, 75.38, 28.59, 26.20, 24.53.; ESI-MS: 497.0 $[M+H]^+$; HRMS(ESI-TOF): *m/z* calculated for $C_{30}H_{32}N_4O_3$ $[M+H]^+$: 497.2547, found: 497.2564.

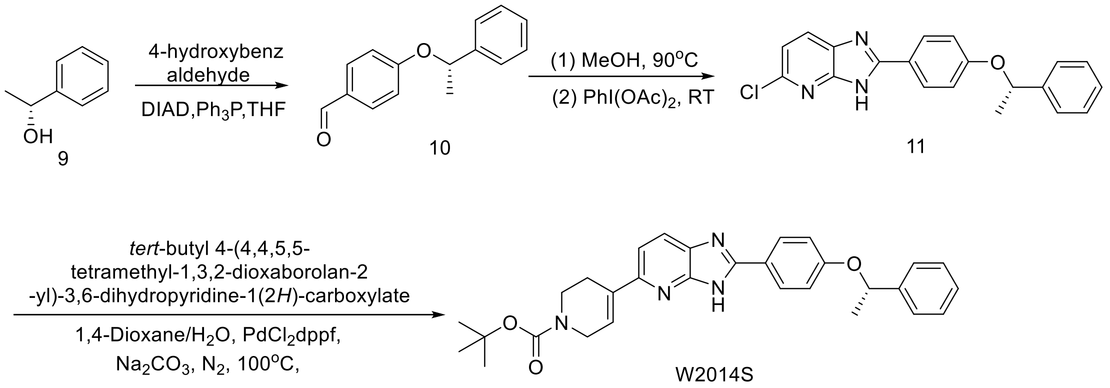

Figure S15. Chemical synthetic route for W2014-R

Step1: Following a similar procedure as that for preparation of compound 7 afforded compound 10 from compound 9 with a yield of 48%. ^1H NMR (400 MHz, $CDCl_3$) δ 9.84 (s, 1H), 7.79 – 7.74 (m, 2H), 7.40 – 7.35 (m, 4H), 7.32 – 7.28 (m, 1H), 6.99 (d, *J* = 8.7 Hz, 2H), 5.44 (q, *J* = 6.4 Hz, 1H), 1.70 (d, *J* = 6.4 Hz, 3H). ESI-MS: 227.2[M+H]+

Step2: Following a similar procedure as that for preparation of compound 8 afforded compound 11 from compound 10 with a yield of 50%. ^1H NMR (400 MHz, $CDCl_3$) δ 11.91(s, 1H), 8.06 – 7.70 (m, 3H), 7.38 (d, *J* = 7.6 Hz, 4H), 7.29 (s, 1H), 7.05 (s, 1H), 6.87 (t, *J* = 40.5 Hz, 2H), 5.40 (d, *J* = 6.1 Hz, 1H), 1.69 (d, *J* = 5.8 Hz, 3H). ESI-MS: 350.8$[M+H]^+$

Step3: Following a similar procedure as that for preparation of W2014 afforded W2014-S from compound 11 with a yield of 61%. ^1H NMR (500 MHz, DMSO) δ 8.07 (d, *J* = 8.1 Hz, 2H), 7.90 (d, *J* = 5.2 Hz, 1H), 7.44 (d, *J* = 7.8 Hz, 3H), 7.36 (t, *J* = 7.3 Hz, 2H), 7.26 (t, *J* = 7.2 Hz, 1H), 7.07 (d, *J* = 8.1 Hz, 2H), 6.63 (s, 1H), 5.63 (d, *J* = 6.2 Hz, 1H), 4.06 (s, 2H), 3.57 (s, 2H), 2.65 (s, 2H), 1.58 (d, *J* = 6.0 Hz, 3H), 1.43 (s, 9H). ^{13}C NMR (126 MHz, DMSO) δ 159.75, 154.44, 151.28, 143.08, 135.60, 129.04, 128.60, 128.00, 126.18, 122.59, 116.59, 114.29, 79.31, 75.39, 62.50, 28.59, 26.20, 25.95, 24.52. ESI-MS: 497.0[M+H]+; HRMS(ESI-TOF): *m/z* calculated for $C_{30}H_{32}N_4O_3$ $[M+H]^+$: 497.2547, found: 497.2563.

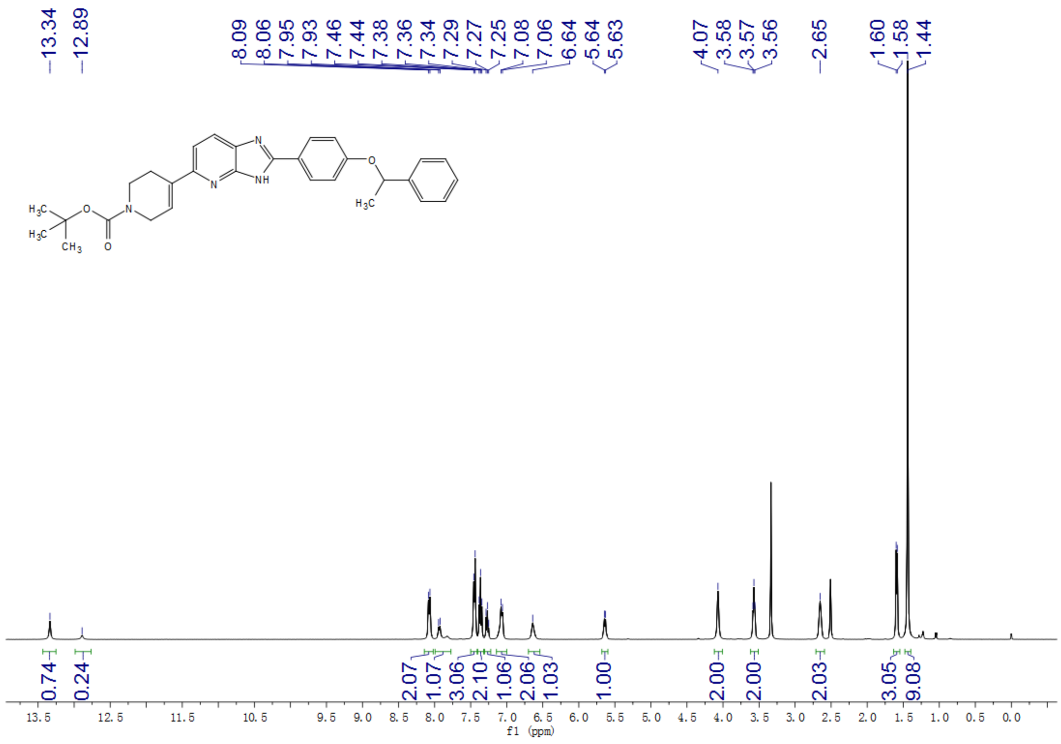

Figure S16. ^{1}H NMR spectrum of W2014

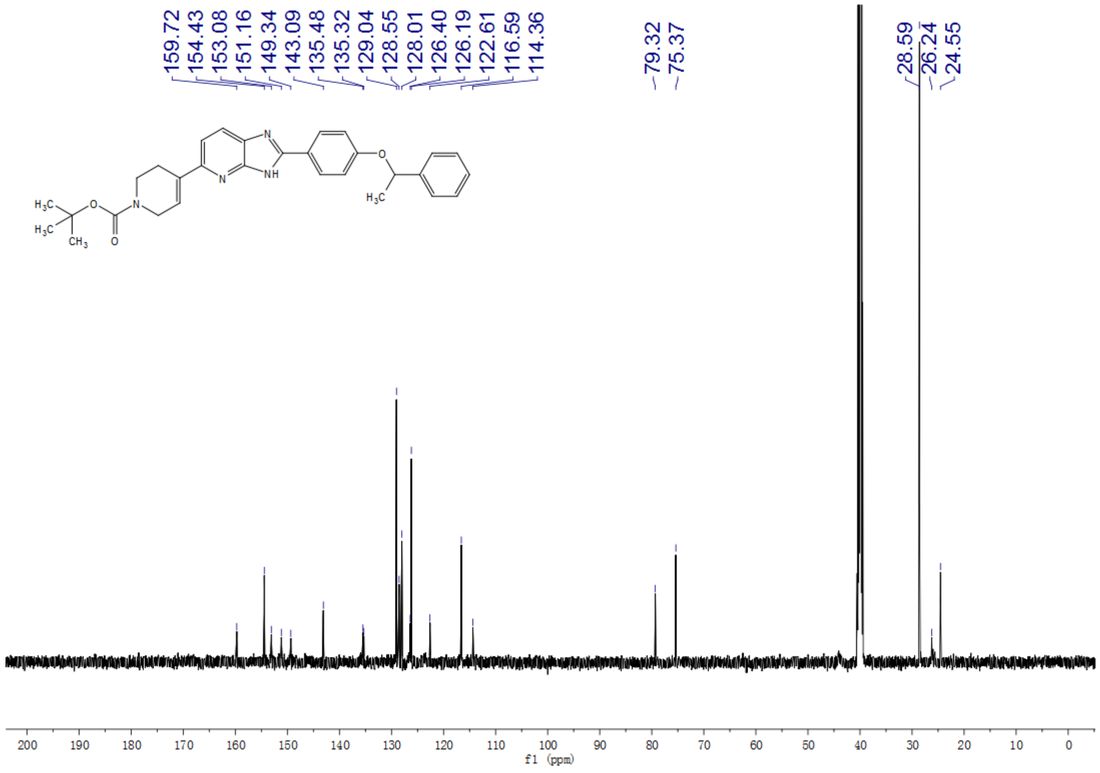

Figure S17. ^{13}C NMR spectrum of W2014

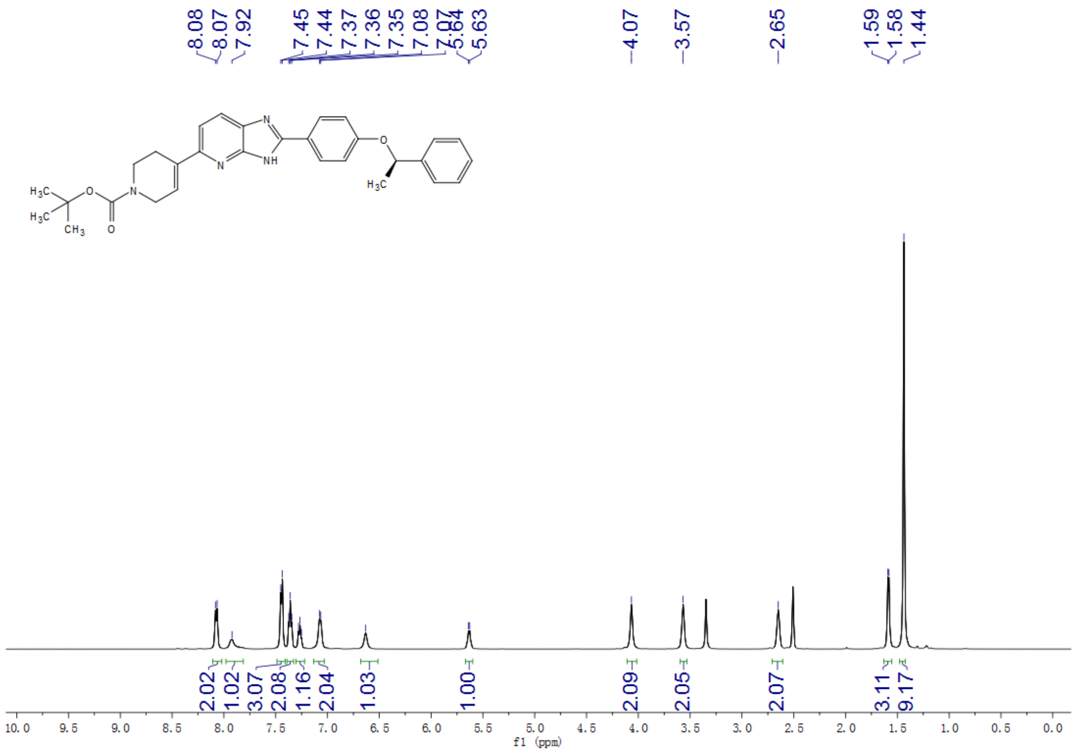

Figure S18. ^{1}H NMR spectrum of W2014-R

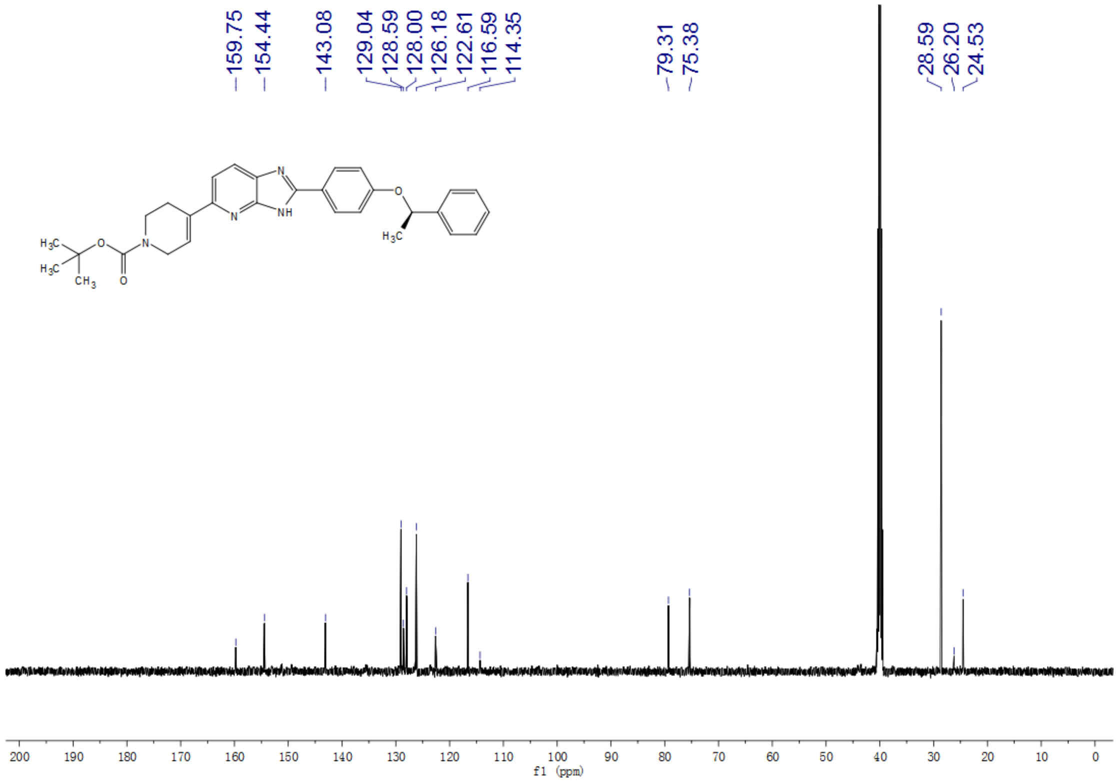

Figure S19. ^{13}C NMR spectrum of W2014-R

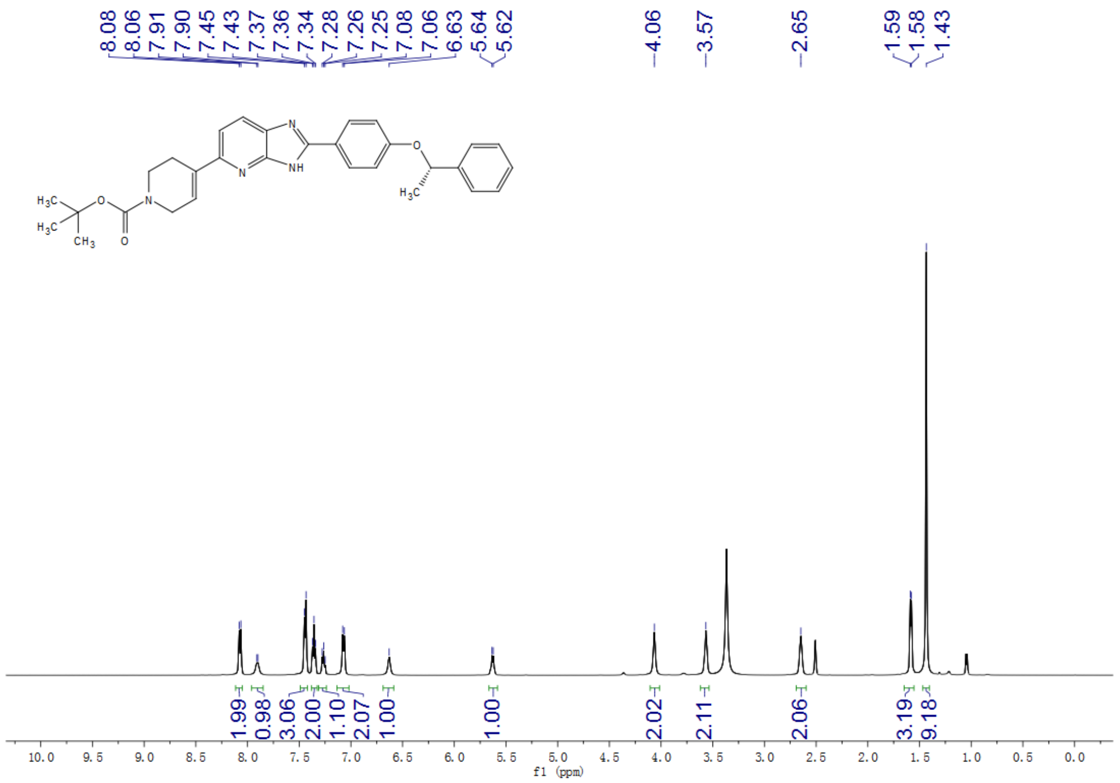

Figure S20. ^{1}H NMR spectrum of W2014-S

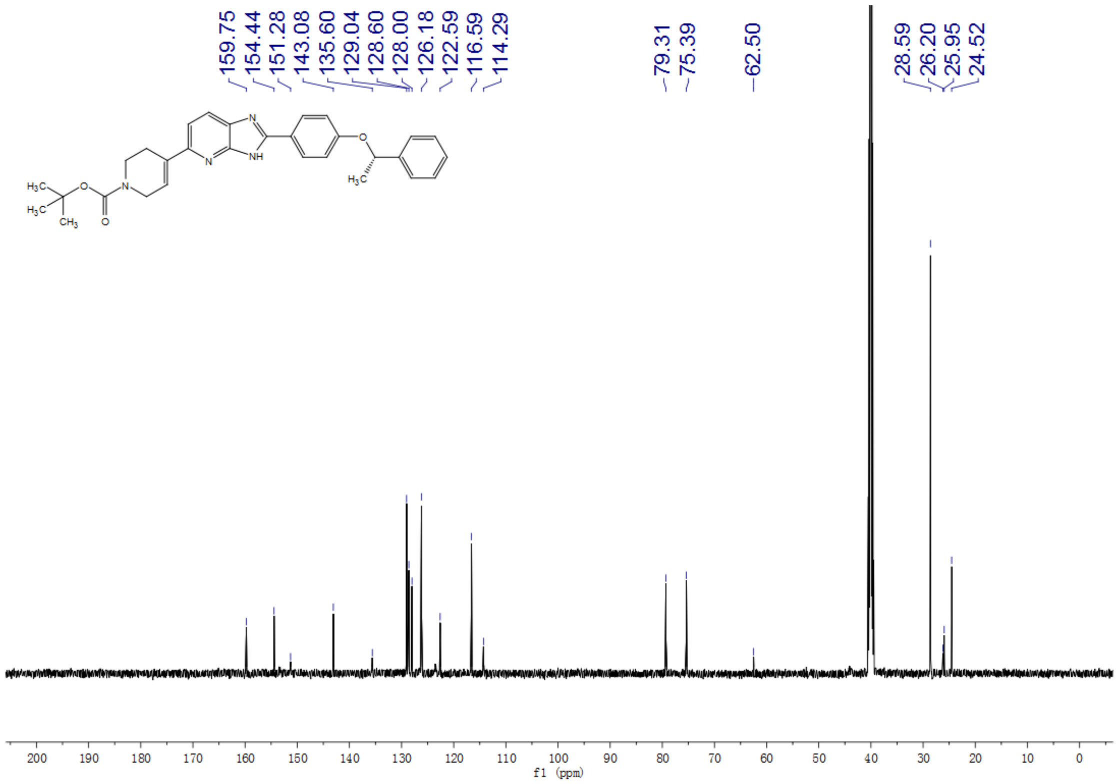

Figure S21. ^{13}C NMR spectrum of W2014-S
